# Systemic effects of rising atmospheric vapor pressure deficit on plant physiology and productivity

**DOI:** 10.1111/gcb.15548

**Published:** 2021-03-08

**Authors:** José López, Danielle A. Way, Walid Sadok

**Affiliations:** ^1^ Department of Agronomy and Plant Genetics University of Minnesota Saint Paul MN USA; ^2^ Department of Biology University of Western Ontario London ON Canada; ^3^ Division of Plant Sciences Research School of Biology Australian National University Canberra ACT Australia; ^4^ Nicholas School of the Environment Duke University Durham NC USA; ^5^ Environmental and Climate Sciences Department Brookhaven National Laboratory Upton NY USA

**Keywords:** climate change, food security, meta‐analysis, plant acclimation, stomatal conductance, vapor pressure deficit

## Abstract

Earth is currently undergoing a global increase in atmospheric vapor pressure deficit (VPD), a trend which is expected to continue as climate warms. This phenomenon has been associated with productivity decreases in ecosystems and yield penalties in crops, with these losses attributed to photosynthetic limitations arising from decreased stomatal conductance. Such VPD increases, however, have occurred over decades, which raises the possibility that stomatal acclimation to VPD plays an important role in determining plant productivity under high VPD. Furthermore, evidence points to more far‐ranging and complex effects of elevated VPD on plant physiology, extending to the anatomical, biochemical, and developmental levels, which could vary substantially across species. Because these complex effects are typically not considered in modeling frameworks, we conducted a quantitative literature review documenting temperature‐independent VPD effects on 112 species and 59 traits and physiological variables, in order to develop an integrated and mechanistic physiological framework. We found that VPD increase reduced yield and primary productivity, an effect that was partially mediated by stomatal acclimation, and also linked with changes in leaf anatomy, nutrient, and hormonal status. The productivity decrease was also associated with negative effects on reproductive development, and changes in architecture and growth rates that could decrease the evaporative surface or minimize embolism risk. Cross‐species quantitative relationships were found between levels of VPD increase and trait responses, and we found differences across plant groups, indicating that future VPD impacts will depend on community assembly and crop functional diversity. Our analysis confirms predictions arising from the hydraulic corollary to Darcy's law, outlines a systemic physiological framework of plant responses to rising VPD, and provides recommendations for future research to better understand and mitigate VPD‐mediated climate change effects on ecosystems and agro‐systems.

## Graphical abstract

Earth is undergoing a global increase in atmospheric vapor pressure deficit (VPD) resulting from “air drying,” the effects of which on ecosystems and agro‐systems are poorly understood. Based on a meta‐analysis of data from 112 plant species across 59 traits, we find that VPD increases systematically reduce crop yields and non‐crop productivity. This reduction, observed under well‐watered conditions, arises from complex effects of VPD on various processes underlying plant physiology, several of which are not taken into account in current modeling efforts. The results point to the need for more research that takes into account this complexity to mitigate future VPD effects.

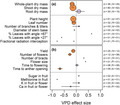

## 1 INTRODUCTION

Accumulating evidence indicates that Earth is currently undergoing a global “atmospheric drying” as a result of an increase in atmospheric water vapor pressure deficit (VPD), a phenomenon that is expected to further amplify as climate change intensifies (Dai et al., 2018; Ficklin & Novick, 2017; Jung et al., 2010; Liu & Sun, 2017; Wang et al., 2012; Yuan et al., 2019). This effect is thought to be driven by two main components: (i) an increase in saturated vapor pressure (the amount of water vapor the air can hold at saturation) as a result of global temperature increases; and (ii) a decrease in actual vapor pressure, which is in part dependent on current air moisture, itself the result of various hydrological processes (Ficklin & Novick, 2017).

Global increases in VPD have been associated with declines in plant productivity worldwide, on crop and non‐crop plants, under a variety of climates (Yuan et al., 2019). At the ecosystem scale, VPD increase has been associated with a decrease in growth and productivity of peatland vegetation (Otieno et al., 2012), grasslands (Ding et al., 2018; Konings et al., 2017), and temperate, mountainous forests (Sanginés de Cárcer et al., 2018). In some cases, VPD effects culminated to trigger tree mortality in drought‐prone environments (Eamus et al., 2013; Will et al., 2013), and limit post‐fire forest seedling recruitment (Davis et al., 2019). In crops, historical increases in VPD have been associated with yield penalties across major agricultural hotspots worldwide, such as the United States, China, and India. In the U.S. Midwest, VPD conditions 60–90 days after sowing were the most important environmental driver of maize yields from 1995 to 2012 in a dataset that also considered temperature and precipitation (Lobell et al., 2014). Furthermore, historical increases in VPD during that period were associated with a slowing of maize yield genetic gains, and even yield decreases. More recently, Mourtzinis et al. (2019) carried out a similar analysis on U.S. soybean and reached a strikingly similar conclusion. A meta‐analysis across the entire U.S. cornbelt region combining the two key crops of maize and soybean indicated a dominant role of VPD over soil moisture in regulating crop productivity (Kimm et al., 2020). Similar findings have been reported in mainland China, where historic VPD increases (1980–2008) were associated with yield decreases of key crops, including wheat, rice, maize, and soybean (Zhang et al., 2017). Even under water‐saturated soil conditions, the VPD increases that occurred across the Indian subcontinent between 1997 and 2008 were associated with significant yield penalties in flooded rice (Tack et al., 2015).

In all of these studies, the mechanistic basis for productivity declines has been linked to photosynthetic limitations arising from decreases in stomatal conductance triggered by rising VPD, either alone or in combination with low soil moisture (reviewed in Grossiord et al., 2020). However, such VPD increases have occurred over decades, which points to the possibility that stomatal acclimation to VPD plays a major role in plant responses to atmospheric drying, yet this effect remains largely overlooked in eco‐physiological, land‐surface and crop models (Grossiord et al., 2020). In addition, a large body of literature points to even more systemic and complex effects of VPD on plant physiology, particularly on the anatomical, biochemical, and developmental levels, independent from variation in soil moisture. For instance, plants exposed to long‐term VPD increases (over weeks to months) exhibit changes in stomatal density and size (e.g., Fanourakis et al., 2013), leaf venation (e.g., Carins Murphy et al., 2014), internal leaf anatomy (e.g., Leuschner, 2002), shoot architecture and root growth (e.g., Darlington et al., 1997; Ford & Thorne, 1974; Gislerød & Nelson, 1989), biochemical composition (e.g., Aliniaeifard et al., 2014; De Luis et al., 2002), and even the growth rate of reproductive organs (e.g., Mortley et al., 2000; Turc et al., 2016).

These wide‐ranging effects, which could impact productivity in a variety of complex ways, are not considered in modeling frameworks aimed at predicting the impacts of atmospheric drying on ecosystems and agro‐systems. This may be a major bottleneck that limits the prospects for more accurately predicting and more effectively mitigating the consequences of VPD increases on plant productivity. Further complicating the matter, the amplitude of these responses may vary as a function of the species, genotype, and experimental setups, while modeling frameworks often build on findings established on a single or a few species.

To address these challenges, here we conduct a quantitative, systematic review of the literature spanning the last five decades (1970–2018), examining longer term (days to years) VPD effects on a vast array of traits and physiological variables (a total of 59) over a large number of plant species (112). While it is well known that stomatal conductance, and hence net CO_2_ assimilation rates, respond to short‐term changes in VPD (i.e., over minutes to hours; e.g., Oren et al., 1999), our focus is on plants acclimated to high VPD conditions. Taking into account potentially confounding environmental effects such as temperature, irrigation frequency, and soil type, our goals are to: (i) identify generalizable VPD response patterns for plant physiology, anatomy, and biochemistry; (ii) extract salient quantitative relationships linking VPD increases and relative changes in key response variables; and (iii) integrate all these responses into a systemic conceptual physiological framework that provides a comprehensive model for understanding long‐term VPD effects on plant productivity. We evaluate some of the most robust findings against parsimonious predictions arising from the hydraulic corollary to Darcy's law, which anticipates substantial changes in global vegetation function and patterns driven by rising VPD effect on vascular function (McDowell & Allen, 2015). We then discuss the implications of these findings and outline recommendations for future research efforts aimed at predicting and mitigating climate change‐driven increases of VPD on food security and ecosystem function.

## 2 MATERIALS AND METHODS

### 2.1 Literature search strategy and selection criteria

The databases Scopus^®^ and Web of Science^®^ were searched between March 30, 2018 and May 13, 2018. The search included the search terms: “VPD,” “vapour pressure deficit,” “vapor pressure deficit,” “evaporative demand,” “acclimation humidity,” “acclimation VPD,” “relative humidity acclimation,” “relative humidity adaptation,” “air humidity acclimation,” “air humidity adaptation,” “stomata humidity,” “air humidity,” “relative humidity,” and “humidity photosynthesis.” These broad searches resulted in a total of 9245 records. The vast majority of the initial records were excluded, as they reflected research themes outside of the scope of the investigation (detailed in Figure S1). The remaining 104 papers addressed the longer term effects of VPD on various plant traits and physiological variables. Effects were considered longer term if these two conditions were fulfilled: (1) the rationale of the study was to investigate longer term effects of VPD (i.e., acclimation); and (2) differential VPD treatments were sustained for 2 days or more.

### 2.2 Data extraction from records

Data extraction from the core 104 papers was undertaken to perform quantitative analyses and enable synthesis of the literature. To perform quantitative analyses, data from each paper were either directly extracted from text and tables or were extracted by digitizing graphs using the online platform WebPlotDigitizer, version 4.1 (https://automeris.io/WebPlotDigitizer). Each record from the 104 papers was scrutinized to extract the following metadata: year of publication, country of origin, species name (as reported in the paper), cultivar or ecotype name (if applicable), type of growth environment (field, greenhouse, growth chamber, room), soil medium (e.g., artificial soil, hydroponics, topsoil mixture, native soil), control and high VPD (kPa), nighttime temperature (T, °C), daytime T (°C), photoperiod (h), photosynthetically active radiation (PAR, μmol m^−2 ^s^−1^), atmospheric CO_2_ concentration (ppm), plant age when the experiment was initiated in days (d), and duration of the VPD treatment (d). Daytime/nighttime VPD could be extracted from most papers (*n* = 98), with the exception of a set of papers from two research groups.

Information about the response of the traits and physiological variables of interest to the VPD treatment was extracted from each paper, leading to the identification of a total of 59 variables (Table 1). We extracted the following information: the trait/variable means observed at each VPD treatment (${\bar{x}}_{h}$ and ${\bar{x}}_{c}$ for high and control VPD, respectively), the sample size ($n_{i}$), and the standard deviation ($s_{i}$). This information was extracted even if the VPD effect was found to be nonsignificant (i.e., *p* > 0.05). Additionally, in the case of gas exchange variables (leaf transpiration rate, stomatal conductance, and net CO_2_ assimilation rate) which were measured with an infrared gas analyzer system, we extracted cuvette conditions (T, VPD, and PAR).

**TABLE 1:** List of 59 traits and physiological variables identified, their descriptions from the original papers, and the corresponding number of studies and species. A study is defined as an experiment carried out on a given species in a paper (i.e., a paper may present multiple studies if it covers multiple species). Units are not reported as they differ widely across studies

Group	Trait and physiological variable name/abbreviation	Short description	No. of studies	No. of species
Leaf gas exchange, development, and anatomy	01. Whole‐plant transpiration rate	Whole‐plant water loss measured gravimetrically	38	21
	02. Leaf transpiration rate	Single‐leaf gas exchange measured via IRGA system	31	25
	03. Stomata conductance	Leaf conductance to H_2_O using IRGA/porometer	62	35
	04. Photosynthetic rate	Single‐leaf gas exchange measured via IRGA system	36	25
	05. Leaf area	Measured on one or more representative leaves	62	36
	06. Leaf expansion rate	Rate of leaf expansion per unit of time	4	2
	07. Leaf dry mass	Mass of oven‐dried leaves	21	11
	08. Specific leaf area	Ratio of leaf area to its mass	37	26
	09. Stomatal size	Stomatal dimensions	27	10
	10. Stomatal density	Stomata number per unit area	37	22
	11. Stomatal index	Number of stomata relative to epidermal cells	15	7
	12. Trichome density	Number of trichomes per unit area	1	1
	13. Epicuticular wax	Amount of leaf epicuticular wax	2	2
	14. Epidermal cell size	Epidermal cell dimensions	9	9
	15. Vein density	Number of veins per unit area	2	2
	16. Leaf thickness	Leaf thickness measured directly by microscope	10	10
	17. Air space fraction in leaf	Area of intercellular air space in the spongy mesophyll	5	5
	18. Spongy mesophyll cell number	Number of spongy mesophyll cells per unit area	2	2
	19. Length of mesophyll cell	Length of the palisade mesophyll cells	4	4
Leaf hormonal, carbohydrate, and mineral status	20. Leaf ABA	Abscisic acid concentration in the leaf	13	6
	21. Leaf starch	Starch content in the leaf	4	3
	22. Leaf soluble carbohydrates	Soluble carbohydrates content in the leaf	4	3
	23. Leaf N	Nitrogen content in the leaf	24	17
	24. Leaf P	Phosphorus content in the leaf	14	6
	25. Leaf K	Potassium content in the leaf	14	7
	26. Leaf Ca	Calcium content in the leaf	20	14
	27. Leaf Mg	Magnesium content in the leaf	7	6
	28. Leaf Fe	Iron content in the leaf	3	2
	29. Leaf Na	Sodium content in the leaf	6	4
	30. Leaf S	Sulfur content in the leaf	3	3
	31. Leaf Zn	Zinc content in the leaf	2	2
	32. Leaf Mo	Molybdenum content in the leaf	1	1
	33. Leaf B	Boron content in the leaf	2	2
	34. Leaf V	Vanadium content in the leaf	2	2
	35. Leaf methionine	Methionine content in the leaf	1	1
	36. Leaf alpha‐aminobutyric acid	Alpha‐aminobutyric acid content in the leaf	1	1
	37. Leaf glutamine	Glutamine content in the leaf	1	1
	38. Leaf threonine	Threonine content in the leaf	1	1
	39. Leaf allothreonine	Allothreonine content in the leaf	1	1
Whole‐plant mass, development, and architecture	40. Whole‐plant dry mass	Total mass of oven dried stems, leaves and roots	36	25
	41. Shoot dry mass	Mass of oven dried stems and leaves	80	62
	42. Root dry mass	Mass of oven dried whole‐roots systems or fine roots	30	15
	43. Plant height	Plant height measured from the collar	61	47
	44. Leaf number	Total number of leaves per plant	32	22
	45. Number of branches and tillers	Total number of branches and tillers per plant	3	3
	46. Diameter of stem base	Measured near the soil	2	2
	47. % Leaves with wide insertion angle	Percentage of leaves with an angle higher than 67°	1	1
	48. % Leaves with narrow insertion angle	Percentage of leaves with an angle lower than 22°	1	1
	49. Fractional radiation interception	Ratio of transmitted to incident short wave radiation	1	1
Yield and reproductive development	50. Yield	Fruit, grain, leaf, root yields of crop plants	25	12
	51. Number of flowers	Number of flowers or flowering buds per plant	12	8
	52. Number of bracts	Number of bracts per plant	1	1
	53. Flower size	Dimensions of flowers	1	1
	54. Time to flowering	Time from the experiment initiation to flowering	8	8
	55. Time to anther opening	Number of hours until most or all anthers are open	9	4
	56. Sugar in fruit	Total sugar content in fruit	2	1
	57. K in fruit or flower	Potassium content in the fruit or flower	3	2
	58. Ca in fruit or flower	Calcium content in the fruit or flower	3	2
	59. Methionine in fruit	Methionine content in the fruit or flower	1	1

Abbreviations: ABA, abscisic acid; B, boron; Ca, calcium; Fe, iron; IRGA, infrared gas analyzer; K, potassium; Mg, magnesium; Mo, molybdenum; N, nitrogen; P, phosphorus; S, sulfur; V, vanadium; Zn, zinc.

To test whether gas exchange acclimation to increasing VPD took place, we distinguished between experimental setups where gas exchange measurements were conducted under the two growth VPD conditions (i.e., low and high), which we labeled “DC” (for different conditions, which assesses gas exchange under the growth conditions), and where these measurements were conducted under the same conditions (labeled SC), after longer term exposure to different VPD conditions (which assesses the degree of acclimation to the growth VPD treatments). The number of observations for DC and SC measurements varied widely as a function of the gas exchange variable, but was comparable for stomatal conductance and photosynthetic rate (see Section 3).

In cases where different species or several cultivars from a single species were examined in the same paper, data were extracted separately for each species or cultivar, and they were referred to as “studies” in the analysis (i.e., a paper may consist of several studies). The number of studies extracted from papers addressing intraspecific diversity in trait and physiological variable response to VPD never exceeded four per paper, which prevented the analysis from being disproportionally influenced by findings arising from a single species. The one exception on this matter was a paper presenting stomatal conductance data from 41 Arabidopsis accessions (Aliniaeifard & van Meeteren, 2014). In this case, we only extracted data from four accessions, which were the only ones that were cross‐examined in multiple experiments in the paper. Finally, in the database, we did not pool data from the same species taken from different papers, as each paper presented a unique set of hypotheses, environmental conditions, and VPD treatments. Overall, the database consisted of 104 papers or records, representing 216 studies covering a total of 112 species (Table S1).

### 2.3 Plant taxonomy and groupings

Since the taxonomy of certain species has changed over the period covered by the publications (1970–2018), the Taxonomic Name Resolution Service v4.0 (TNRS, http://tnrs.iplantcollaborative.org/TNRSapp.html) was used to update the scientific names when needed and to identify accepted naming authorities and botanic families. For our analyses, we organized the species into different groups based on evolutionary history (dicot, fern, gymnosperm, or monocot), growth habit (forb, woody, and grass), growth duration (annual or biennial, or perennial), and end‐use (crop vs. non‐crop). These plant groupings were assigned based on the PLANTS database of the USDA Natural Resources Conservation Service (https://plants.sc.egov.usda.gov/classification.html).

### 2.4 Data analysis

#### 2.4.1 Mean VPD effect size and confidence intervals

Data analysis was undertaken to synthesize the literature and visualize patterns based on the meta‐analysis. First, we computed the response ratio (R), as follows:

(1) $$R=\frac{{\bar{x}}_{h}}{{\bar{x}}_{c}},$$

where ${\bar{x}}_{h}$ is the mean value for the trait in the high VPD treatment and ${\bar{x}}_{c}$ is the mean of the trait in the control VPD treatment. Since the response ratio is non‐normal and nonlinear, we conducted all the analyses on the natural logarithm of R (i.e., L). This variable was chosen over R because it is equally affected by changes in the numerator or denominator and is more normally distributed in small samples (Hedges et al., 1999).

Second, based on the sample size ($n_{c}$ and $n_{h}$, for control and high VPD treatments, respectively) and the standard deviation for ${\bar{x}}_{c}$ (labeled $s_{c}$) and ${\bar{x}}_{h}$ (labeled $s_{h}$) reported in each study, we approximated the variance (v) of L as follows (Hedges et al., 1999):

(2) $$v=\frac{{\left(s_{h}\right)}^{2}}{n_{h}{\bar{x}}_{h}}+\frac{{\left(s_{c}\right)}^{2}}{n_{c}{\bar{x}}_{c}}.$$

Subsequently, we used this information to estimate the weighted mean of L across studies ($L^{*}$), following Hedges et al. (1999). While we were able to estimate $s_{c}$ and $s_{h}$ for most studies (66%), the rest did not report standard deviations or any statistic that could be used to derive them. In such cases, we imputed $s_{c}$ and $s_{h}$ based on the average coefficient of variation across all studies with non‐missing data for the trait of interest, following He and Dijkstra (2014) and Bai et al. (2013). However, for three traits (air space fraction in leaf, length of mesophyll cell, and time to anther opening), all of the studies had missing $s_{i}$. In these cases, we assigned each observation of $L_{i}$ a weight of 1, as suggested in Jablonski et al. (2002) and Marty and BassiriRad (2014).

Third, we calculated the bootstrap 95% confidence intervals (CIs) using the approach described by Adams et al. (1997). Briefly, for each trait, we chose i studies at random with replacement and calculated $L^{*}$, repeating this process 4999 times. We used the bias‐corrected accelerated CI because this method is more robust for smaller values of i (Adams et al., 1997; Efron, 1987). If i was smaller than 3, we did not calculate CIs and reported only $L^{*}$. Finally, the results, that is $L^{*}$ and its CIs, were transformed back to R (antilog of $L^{*}$) and converted to a VPD effect size (R‐1) since this value is easier to interpret.

#### 2.4.2 Mixed model meta‐regression analyses

In cases where traits and physiological variables were measured over a large number of studies (*n* > 20), analyses were carried out to quantify the effect of VPD change on these response variables and estimate the extent to which this relationship was influenced by potentially confounding environmental factors (i.e., moderator variables). The level of VPD change was expressed as a VPD ratio as follows:

(3) $$\text{VPD}\;\text{ratio}=\frac{{\text{VPD}}_{h}}{{\text{VPD}}_{c}},$$

where ${\text{VPD}}_{h}$ and ${\text{VPD}}_{c}$ represent the VPD conditions in the high VPD and control treatments, respectively.

Other than the VPD ratio, the considered moderators were: irrigation frequency (two categories: hydroponic/daily, less‐than‐daily/no information), soil type (five categories: artificial soil, hydroponic medium, native soil, topsoil mixture, unspecified) and daytime temperature. These were chosen to account for the well‐documented dependency of VPD effects on soil moisture availability and temperature (e.g., Bouchabké et al., 2006). Furthermore, we used treatment duration and plant age (continuous variables: days) as additional moderators. The VPD ratio was log‐transformed because, similar to the response ratio (R), the untransformed ratio is highly sensitive to changes in the denominator and is non‐normal and nonlinear. While all papers reported that plants were not exposed to irrigation deficit during the experiments, in the case of irrigation frequency, we extracted descriptors distinguishing between studies where plants were watered daily or grown in a hydroponic system (Group 1) and those where plants were reported as “well‐watered” with no further details (Group 2).

We evaluated the effect of these moderators on the natural logarithm of the response ratio (L) using mixed model meta‐regression (Gilbert et al., 2011; Hedges et al., 2010). To determine which moderator variables significantly explained differences in L across studies, we followed the minimal adequate model approach described by Crawley (2015). This approach consists of removing nonsignificant parameters (in this case moderators with *p*‐values higher than 0.05) one by one until only significant parameters are left in the model. The initial model, or full model, contained all the moderators listed above.

Subsequently, we evaluated whether differences in VPD effect size could be related to differences across botanical families and the four considered plant groupings (e.g., growth habit). Since a multiple meta‐regression analysis with these categorical moderators would capture redundant dummy variables in the model, a single factor meta‐regression approach was used to evaluate one moderator at a time.

All analyses were implemented in R (R Core Team, 2016), as follows. Bootstrap CIs were calculated using the R package boot v. 1.3.20 (Canty & Ripley, 2008), mixed‐model meta regressions were implemented in the r‐package metafor (Viechtbauer, 2010), data from Excel were read into R using the package xlsx v. 0.6.1 (Dragulescu, 2014), and color‐blind‐friendly palettes in the figures were generated with the aid of the viridis v. 0.5.1 package (Garnier, 2018).

## 3 RESULTS

### 3.1 Diversity of species and environmental conditions covered by the meta‐analysis

**FIGURE 1:**
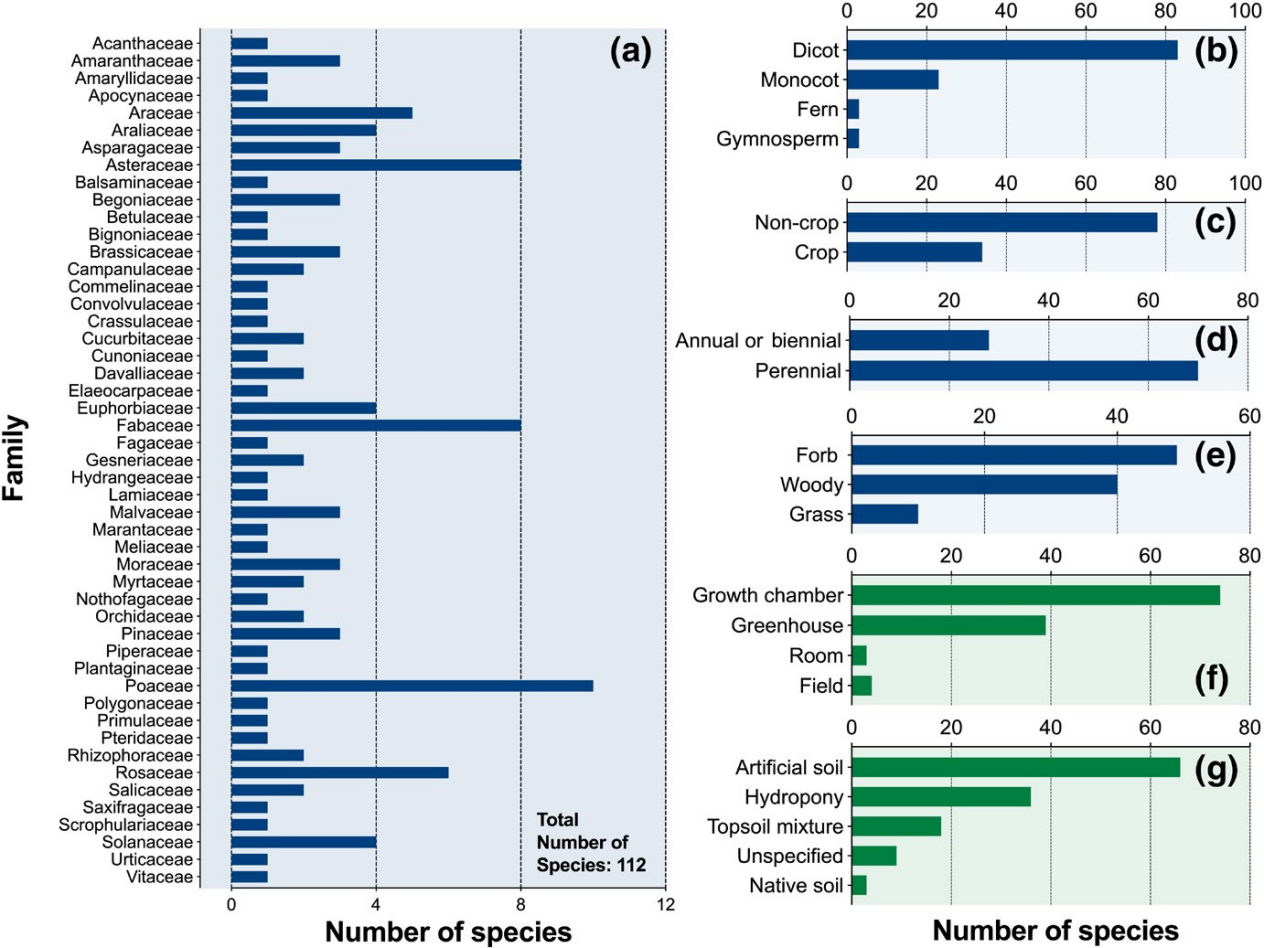
Diversity of the plants examined in the study. (a) Botanical families and number of plant species per family. (b–g) Distribution of the number of species as a function of plant and environment types. In the case of (d) and (e), the growth duration (i.e., annual, biennial, or perennial) (d) and habit (e) were not available for 14 species

In total, the meta‐analysis covered 112 species and 49 families, with Asteraceae (8 species), Fabaceae (8 species), and Poaceae (10 species) being the most frequently represented groups (Figure 1a; Table S1). The vast majority of species (84%) exhibited a significant response to VPD for one or more of the examined response variables, while a subset of 18 species was not responsive to VPD. Most of these VPD‐insensitive species (15 out of the 18 species) were from the same paper (Mortensen & Gislerød, 1990), and although this group represents a diverse set of species (11 families), they were mostly perennial forbs or trees.

**FIGURE 2:**
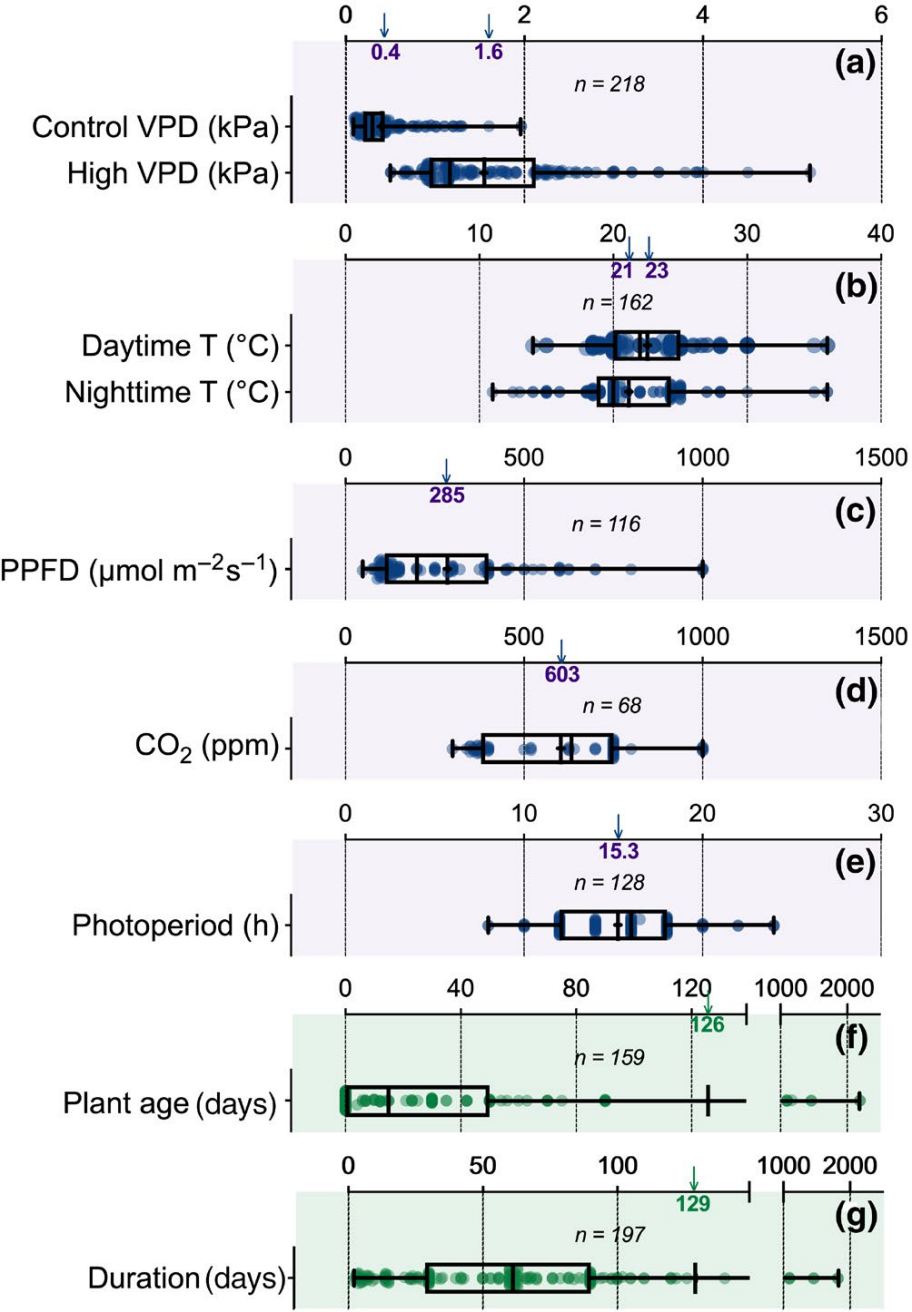
Ranges of average environmental conditions (a–e), plant age when the experiment was initiated (f), and treatment duration (g) for all studies considered in the meta‐analysis. In each panel, *n* represents the number of studies. Box and whiskers and scatter plots represent the range of each environmental variable. In the box and whiskers, the box represents the median and the 25th–75th percentiles while the whiskers indicate minimal and maximal values. The “+” sign represents the mean value for the considered variable. In each panel, this mean value is reported on the *x*‐axis by the vertical arrow and the number at the bottom of the arrowhead. PPFD, photosynthetic photon flux density; T, temperature; VPD, vapor pressure deficit

As shown in Figure 1b–g, most of the species examined for VPD responses were dicots (74%), perennial (71%), non‐crops (70%), and forb or woody plants (90%). The majority of species were examined under controlled (growth chamber) to semi‐controlled (greenhouse) conditions (97%), with most plants grown in hydroponics or artificial soil (77%). Across studies, average control and high VPD values strongly segregated around mean values of 0.4 and 1.6 kPa, respectively (Figure 2a), while mean day and night T were ~23°C and 21°C, respectively (Figure 2b). Overall, responses to increases in VPD were observed under wide environmental gradients (Figure 2c–e) for irradiance (48–1000 μmol m^−2^ s^−1^), atmospheric CO_2_ concentration (300–1000 ppm), and photoperiod (8–24 h), although these environmental conditions were held constant across VPD treatments within each study.

### 3.2 Diversity and patterns in trait responses to VPD

A total of 59 traits and physiological variables were reported to be significantly influenced by VPD in at least one of the records (Table 1). These variables encompass processes operating at different organizational levels from the cell to the whole plant, including tissue anatomy, gas exchange, nutrient and hormonal status, organ growth and development, whole‐plant architecture, reproductive success, and agronomic yield.

**FIGURE 3:**
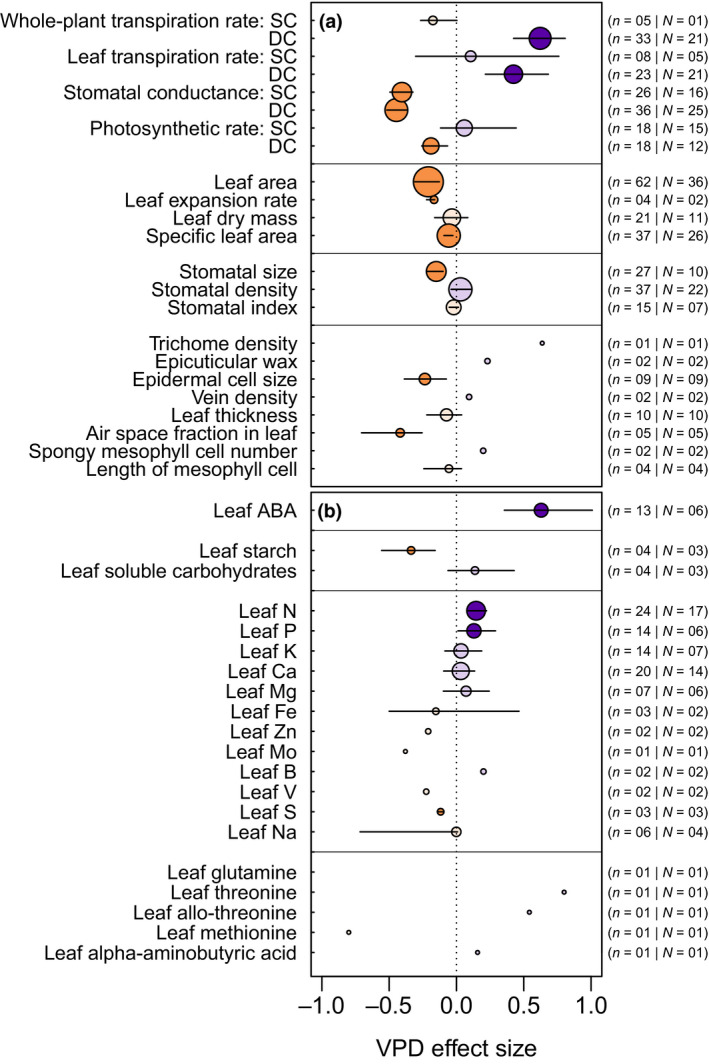
Vapor pressure deficit (VPD) effect size for leaf gas exchange, development, and anatomy (a), and leaf biochemical status (b). Horizontal lines represent 95% confidence intervals (CI). Because these are bias‐corrected bootstrap intervals, the intervals are on occasion not centered around the estimated value. CI lines are not drawn when the number of studies is lower than 3. Purple and orange colors reflect VPD effect size values higher or lower than zero, respectively. Traits and physiological variables with 95% CI not intersecting with zero are highlighted with darker shades. The numbers reported on the right‐hand side, that is, *n* and *N*, represent the total number of studies and number of species used, respectively. In panel (a), the abbreviations SC and DC represent conditions during gas exchange measurements (SC, same condition; DC, different conditions; see Section 2 for details). Data for traits and physiological variables with an effect size over 1 or under −1 are omitted to maximize comparability. This is the case for glutamine (*n* = 1, effect size = 1.97). ABA, abscisic acid; B, boron; Ca, calcium; Fe, iron; K, potassium; Mg, magnesium; Mo, molybdenum; N, nitrogen; P, phosphorus; S, sulfur; V, vanadium; Zn, zinc. See Table 1 for trait descriptions

**FIGURE 4:**
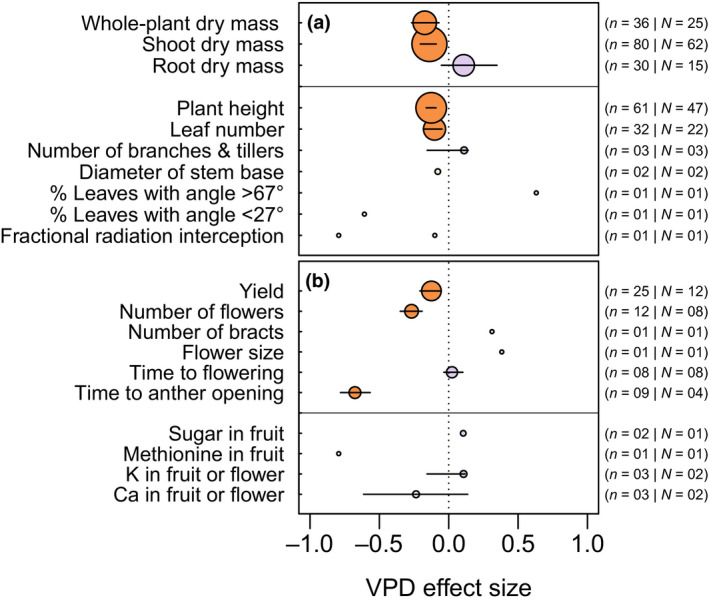
Vapor pressure deficit (VPD) effect size for traits related to whole‐plant dry mass, development, and architecture (a), and yield and reproductive development (b). The components of the figure are as described in the caption for Figure 3

Combining the quantitative data extracted from these records, and despite the substantial diversity in species, experimental setups, and growth conditions (Figures 1 and 2), general patterns emerged (Figures 3 and 4), particularly for traits examined over a large number of studies (*n*) and species (*N*). This is particularly the case for traits and physiological variables such as whole‐plant transpiration rate (*n* = 38, *N* = 21), stomatal conductance (*n* = 62, *N* = 35), leaf area (*n* = 62, *N* = 36), whole‐plant dry mass (*n* = 36, *N* = 25), shoot dry mass (*n* = 80, *N* = 62), and plant height (*n* = 61, *N* = 47), all of which responded significantly to increased VPD. In terms of effect on gas exchange, exposure to elevated VPD increased transpiration, decreased stomatal conductance, and led to a relative decrease in photosynthesis (Figure 3a). However, a clear distinction could be made between measurements made under the same and different cuvette conditions (SC and DC, respectively), particularly for stomatal conductance and photosynthesis. When leaves were measured at the same cuvette conditions (SC), plants exposed to high VPD during growth had lower stomatal conductance relative to plants exposed to the control VPD, although net CO_2_ assimilation rates were similar between the two sets of plants. In contrast, when leaves were measured under their different respective treatment VPDs (DC), plants from the higher VPD treatment showed both lower stomatal conductance and net CO_2_ assimilation rates than their counterparts from the control VPD treatment. In the case of whole‐plant and leaf‐level transpiration rates, SC measurements were not significantly impacted by growth VPD, which may be attributable to the limited number of observations available (Figure 3a), or to researchers using a common VPD for the airstream entering the gas exchange cuvette, which could lead to a lower cuvette VPD in leaves with low stomatal conductance (i.e., the high VPD‐grown plants), and thus an increase in transpiration in these samples. For these same variables, DC measurements were associated with an increase in transpiration as treatment (and measurement) VPD increased.

In terms of leaf growth and anatomy, the higher VPD treatment generally decreased leaf area, leaf expansion rates, and specific leaf area, though there was no significant effect of treatment VPD on leaf dry mass (Figure 3a). Additionally, longer term exposure to high VPD decreased stomatal size, epidermal cell size, and the air space fraction inside the leaf. Despite the large number of observations (*n* = 37, *N* = 22), results from studies examining stomatal density were highly divergent, resulting in this variable being nonsignificantly affected by VPD, which echoes the results for stomatal index (Figure 3a). In contrast, the effects of VPD increase on leaf hormonal and mineral content generally led to higher values in these parameters (Figure 3b), including an increased accumulation of abscisic acid (ABA) and mineral nutrients, particularly N and P. While K, Ca, and Mg concentrations tended to increase in high VPD‐treated plants, these changes were not significant. Other mineral nutrient concentrations (Zn, Mo, B, V, and S) were also significantly altered by treatment VPD, though few studies assessed these parameters. There was some evidence for changes in leaf amino acid concentrations, though this is based on a small number of studies. For leaf carbohydrates, longer term exposure to high VPD reduced starch levels, but tended to increase soluble carbohydrate concentrations.

At the organismal level, high treatment VPD reduced shoot and whole‐plant growth (Figure 4a). Overall, there was no significant tendency for root mass to vary in response to treatment VPD (Figure 4a). Most aboveground architectural traits were impacted by longer term exposure to high VPD, reflecting a decrease in plant height, leaf number, and stem diameter. In terms of reproductive growth and development, increased VPD led to a lower number of flowers (Figure 4b), a strong reduction in the time needed for anther opening, potential changes in fruit/flower composition, and perhaps most importantly, significant yield penalties (Figure 4b).

### 3.3 Moderator effects on VPD effect size and emergent relationships

For the vast majority of the traits and physiological variables covered by the mixed model meta‐regression (13 out of 16), the analysis revealed that VPD effect size was not influenced by soil water availability (approximated through irrigation frequency; *α* = 0.05), with the exception of whole‐plant and leaf‐level transpiration rates, and leaf number. For whole‐plant and leaf transpiration rates, studies that reported less than daily irrigation frequencies or did not report irrigation frequency had a 70% and 76%, respectively, lower VPD effect size than studies with hydroponics or daily watering (*p* < 0.01 and <0.05, respectively). Thus, transpiration in plants from experiments that minimized root water stress was more sensitive to high VPD treatments, indicating that the true VPD effect size on these traits and physiological variables may have been underestimated because of the lower irrigation frequency in some studies. The opposite was found for VPD effect size on leaf number, where studies with less frequent or undefined irrigation frequencies had a higher VPD effect size than studies with ample water (*p* < 0.05), such that the true effect of VPD on leaf number was potentially overestimated across the whole dataset (*p* < 0.05).

Variation in temperature across studies significantly affected the VPD effect size for SLA and stomatal density, with the effect of temperature increase counteracting the effect of VPD on SLA, while amplifying the VPD effect on stomatal density (*p* < 0.05). None of the 16 traits exhibited a significant dependency of VPD effect size on soil type or treatment duration. For two traits (leaf N and yield) plant age significantly impacted the VPD effect size (*p* < 0.01 for both), with older plants tending to offset the VPD effect (reported in Figures 3 and 4) on both traits.

**FIGURE 5:**
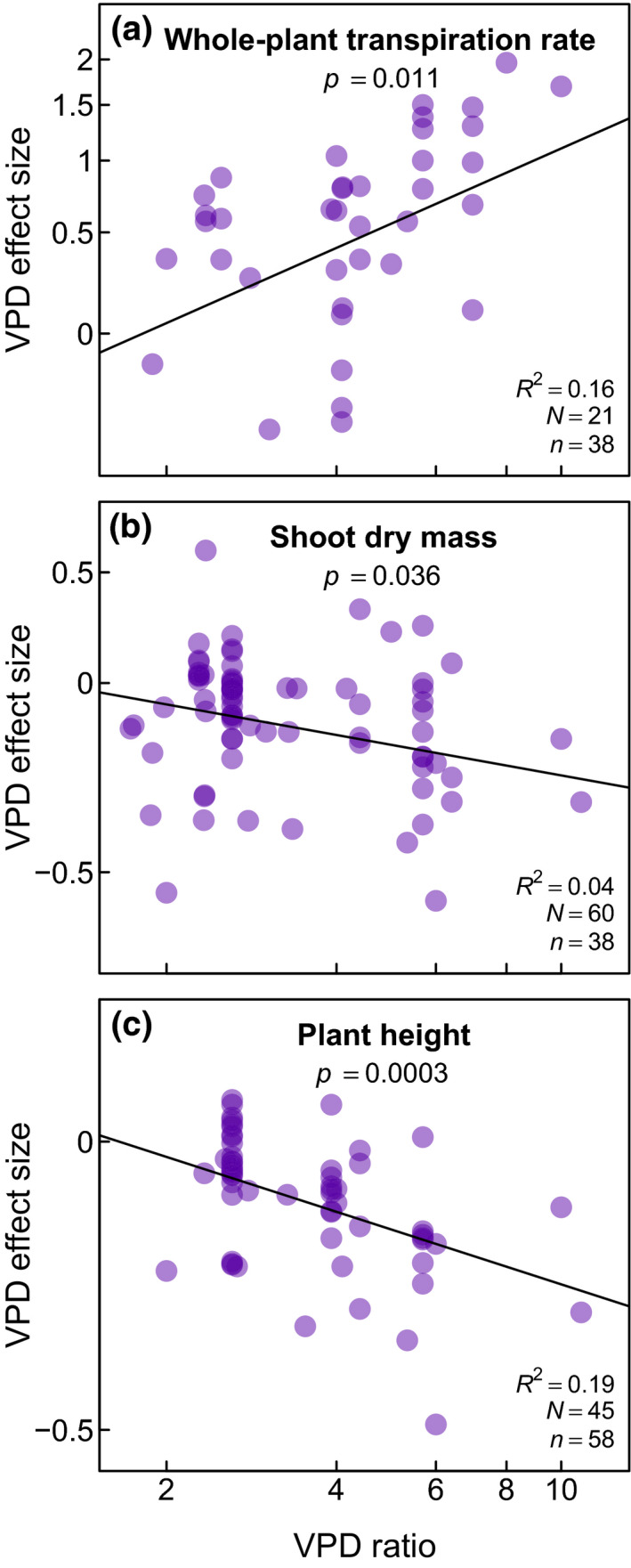
Significant relationships (*p* < 0.05) between vapor pressure deficit (VPD) effect size and VPD ratio, both expressed on a logarithmic scale (see Section 2 for details). Letters *N* and *n* refer to the numbers of species and studies, respectively

The VPD ratio strongly correlated with VPD effect size for three traits and physiological variables: whole‐plant transpiration rate, shoot dry mass, and plant height (Figure 5). In these relationships, the size of the VPD effect on whole‐plant transpiration rate increased proportionally with VPD ratio (Figure 5a), indicating that a larger increase in VPD led to a greater increase in transpirational water loss. The opposite trend was found for shoot dry mass (Figure 5b) and plant height (Figure 5c), such that greater increases in VPD produced stronger decreases in shoot height and mass.

### 3.4 VPD effect size as a function of plant life history strategies and botanical families

**FIGURE 6:**
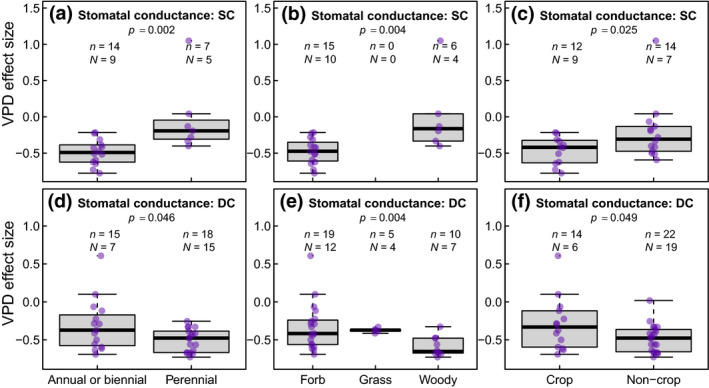
Significant differences in vapor pressure deficit (VPD) effect size as a function of plant functional types for stomatal conductance measured under same or different cuvette conditions (SC and DC, respectively). Individual datapoints are plotted along the box and whiskers to enable visualization of outliers. The thick line in the center of each box and whisker represents the median, while the box represents the interquartile range (IQR, i.e., the 25th–75th percentile), with the whiskers extending to values that are 1.5 times the value of IQR. Letters *N* and *n* refer to the numbers of species and studies, respectively

**FIGURE 7:**
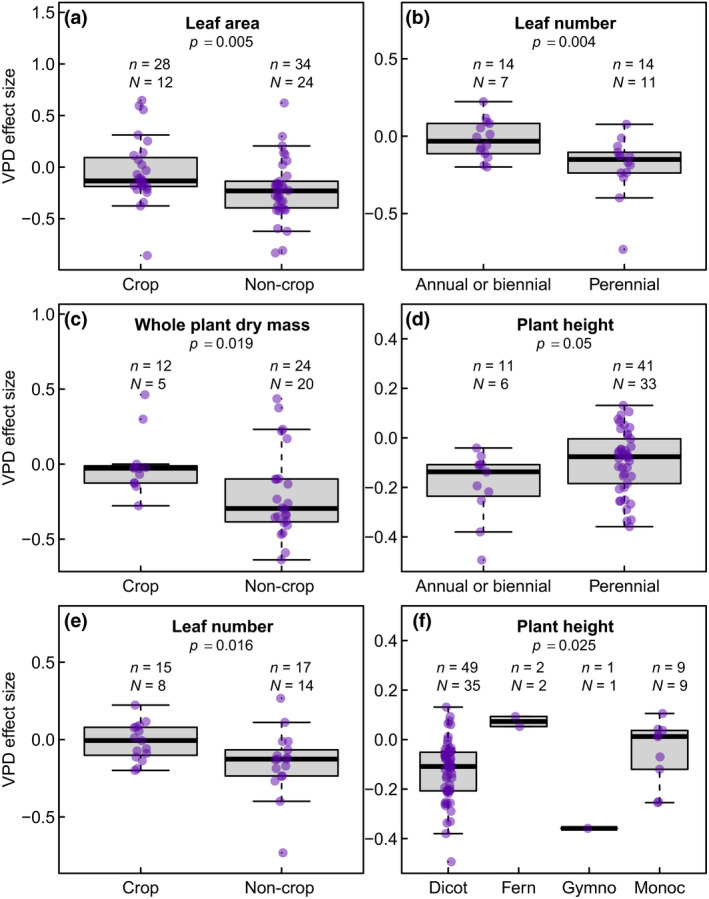
Plant traits (a–f) exhibiting significant (*p* < 0.05) or near‐significant variation (*p* = 0.05) in vapor pressure deficit (VPD) effect size as a function of plant functional types. The other attributes of the figure are as described in the caption of Figure 6

For response variables measured across a large number of species (*N* > 20; i.e., whole‐plant transpiration rate, leaf transpiration rate, stomatal conductance, leaf area, specific leaf area, stomatal density, whole‐plant dry mass, shoot dry mass, plant height, and leaf number), the mixed model meta‐regression analysis enabled the detection of differences in VPD effect size as a function of the four typologies of plant groupings considered in the study. These effects varied widely as a function of the trait and the moderator being considered (Figures 6 and 7). For gas exchange, the VPD effect size on whole‐plant transpiration rate was not influenced by any of the four groupings. However, the VPD effect size for leaf transpiration rate was significantly influenced by plant end‐use (crop vs. non‐crop), where non‐crop plants exhibited a stronger, positive VPD effect size compared to crop plants (*p* = 0.019, data not shown). However, the limited number of observations for SC and DC data for this variable (see Figure 3) prevented a comparative analysis of SC and DC data.

For stomatal conductance, significant differences between groups differing in growth duration (annual/biennial vs. perennial), growth habit (forb vs. grass vs. woody), and end‐use (crop vs. non‐crop) emerged as a function of conditions during measurements (Figure 6). Under the SC measurements, annual/biennial species (Figure 6a), forbs (Figure 6b), and crops (Figure 6f) exhibited a stronger reduction in stomatal conductance as a result of VPD increase compared to perennial, woody, and non‐crop plant species, respectively, indicating a stronger acclimation to the high VPD treatments. In contrast, the exact opposite trend was observed when stomatal conductance was measured under the DC treatment. Under this DC treatment, halophytic species grown in seawater (*n* = 6, *N* = 4) also showed a stronger decrease in stomatal conductance (*p* = 0.02) compared to non‐halophytic species (*n* = 30, *N* = 21, data not shown).

For the rest of the variables, the most widespread discriminator among plant groupings was end‐use (i.e., crop vs. non‐crop, Figure 7a,c,e). Specifically, non‐crop plants tended to exhibit a stronger response to increases in treatment VPD, particularly for leaf area, whole‐plant dry mass, and leaf number, which were reduced more strongly under longer term high VPD conditions than in crop species (Figure 7a,c,e). Perenniality was found to be another significant moderator, specifically for leaf number and plant height (Figure 7b,d). In this case, perennial species exhibited a stronger decrease in leaf number in response to increases in VPD, but a weaker decrease in plant height, in comparison to annual/biennial plants. Across plant groupings representing evolutionary history (Figure 7f), gymnosperms exhibited the strongest reduction in plant height, while ferns expressed a positive response of plant height to increasing VPD. However, these observations have to be weighted by the fact that the number of measurements available was particularly low for ferns (*n* = 2) and gymnosperms (*n* = 1).

## 4 DISCUSSION

**FIGURE 8:**
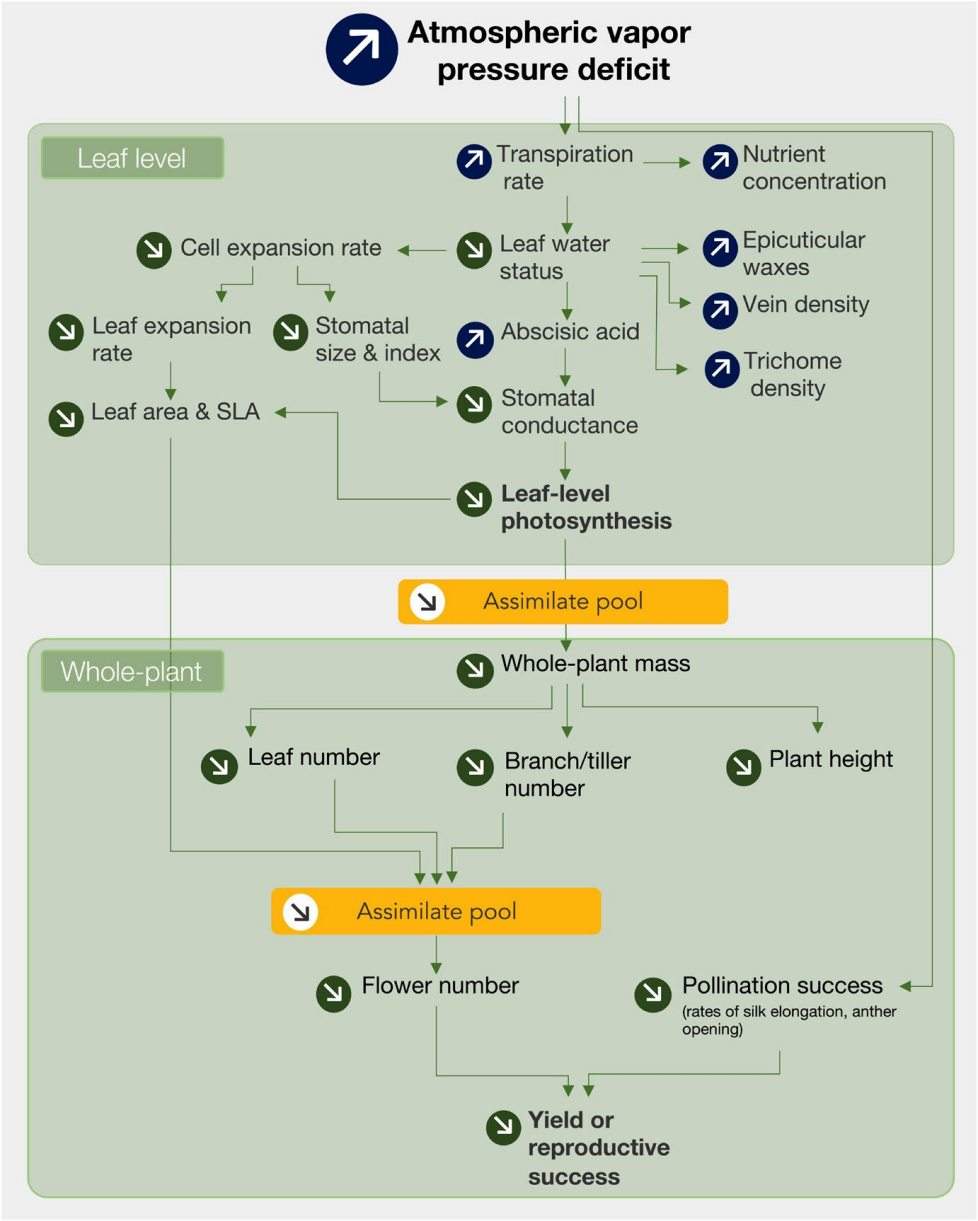
A general framework synthesizing the integrated effects of atmospheric vapor pressure deficit (VPD) increase on plant productivity and yield, based on the meta‐analysis. Arrows in circles represent the direction of change in the trait (increase or decrease) as a result of increased VPD. The two main organizational scales (leaf and whole‐plant) are separated in two green boxes for clarity. Green arrows depict relationships between traits within and across organizational scales, which were identified based on the literature review. Orange boxes refer to leaf‐level and whole‐plant‐level photoassimilate (carbon‐based) pools. Abbreviations are explained in Table 1

The hydraulic corollary of Darcy's law predicts that future increases in atmospheric VPD will favor the survival of vegetation that is shorter and equipped with a smaller evaporative surface (McDowell & Allen, 2015). The broad strokes of our meta‐analysis support this prediction (Figures 4 and 5 and below sections), while revealing that over the long term, an increase in atmospheric VPD will generate more complex and systemic effects on vegetation than previously thought (Figure 8), that is, on multiple organizational levels (cell to whole‐plant) and tissue types (leaves, shoots, roots, and reproductive organs). As predicted by the corollary of Darcy's law, a rise in VPD had a particularly strong impact on response variables capturing processes that drive the global water cycle (i.e., transpiration and stomatal conductance), and carbon fixation (i.e., photosynthesis and canopy growth, Figure 3). But it also emerged that while plants acclimate to increasing VPD, there are still major costs of growth at high VPD, leading to changes in plant N status and reductions in primary productivity and crop yields (Figures 3 and 4). Importantly, all of these effects were observed in well‐watered and nonsaline hydroponic studies, indicating that future increases in atmospheric drought, even in the absence of greater soil water stress, will reduce plant growth and alter biogeochemical cycling. Our data give strong biological support to the recent observation that a worldwide decline in plant productivity has been taking place, independently of water availability regimes, as a result of a global increase in VPD since the 2000s (Yuan et al., 2019).

Additionally, the evidence assembled here points to differences in responses among plants with different life history strategies, with individual studies also showing intraspecies variation, pointing to the possibility of mitigation through management of plant community assembly or breeding (for crop plants). Below, we synthesize the key emerging mechanisms to high VPD growth conditions and discuss their implications as a basis for future research.

### 4.1 Stomata acclimate to longer term VPD increase

Our data show that elevated VPD during growth leads to higher rates of transpirational water loss. Consequent reductions in leaf water status will decrease stomatal conductance and thereby reduce CO_2_ capture, an observation that is consistent with the mechanistic basis of stomatal response to leaf water status (Buckley, 2005; Peak & Mott, 2011). Unfortunately, evidence of acclimation could not be inferred from the data available to us on transpiration. However, for stomatal conductance, we had sufficient information to consider both SC data (which measure acclimation to the treatment) and DC data (which assess performance under the treatment conditions, combining both acclimation and short‐term, acute responses to changes in VPD). The SC stomatal conductance data indicate that longer term exposure to increasing VPD leads to a decrease in stomatal conductance, likely linked to developmental changes that led to the formation of smaller stomata (Figure 3a). In the SC measurements, this decrease in stomatal conductance does not suppress photosynthesis, likely as a result of increased leaf N concentrations (Figure 3b), which implies that plants offset their lower CO_2_ supply rate via an increase in photosynthetic capacity. In the DC data, the combination of a high treatment and measurement VPD leads to a further decrease in stomatal conductance than in the SC data, and a substantial suppression of photosynthesis.

The relatively small difference in stomatal conductance between the SC and DC data implies that high treatment VPD may reduce stomatal sensitivity to VPD. This is consistent with work showing that stomatal sensitivity to VPD correlates with stomatal conductance measured at a reference VPD of 1 kPa; leaves with lower stomatal conductance at 1 kPa show less stomatal sensitivity to increasing VPD (Oren et al., 1999; Whitehead & Jarvis, 1981). As indicated in Figure 6, this acclimation effect was not randomly distributed across plant groupings. The distribution indicates that annual plants, and particularly crops, exhibit a stronger stomatal acclimation response to VPD, which could indicate that artificial selection is already favoring phenotypic plasticity for this response as a way to enhance crop resilience toward water deficits. Additionally, the patterns in our gas exchange data are also in line with the expectation that photosynthesis will be more strongly suppressed by a reduction in stomatal conductance when the latter is already low, given the nonlinear, saturating response of net CO_2_ assimilation rates to stomatal conductance (Farquhar & Sharkey, 1982).

### 4.2 An integrated developmental, hormonal, and nutritional response to VPD increase

Consistent with the hydraulic corollary of Darcy's law, increases in VPD during growth were also associated with slower growth rates and decreased vegetative phytomass (Figures 3a and 4a). In addition, at the leaf level, decreased growth rates were correlated with anatomical, hormonal, and nutrient composition changes (Figure 3a,b). These changes appear to reflect an acclimation strategy at the leaf level, where increased VPD during growth increases transpiration, triggering a decrease in leaf water potential. This, in turn, leads to ABA accumulation in the growing leaves, thereby priming the leaf to adjust its evaporative surface by reducing leaf area, stomatal size, and mesophyll airspace (Figure 3a). While these relationships were not all observed within a single study, they are consistent with literature documenting effects of ABA accumulation, alone or in interaction with hydraulic signals, on reducing leaf expansion rate (Salah & Tardieu, 1997), stomatal size (Franks & Farquhar, 2001), intercellular leaf airspace (Severi & Fornasiero, 1983; Young et al., 1990), and mesophyll conductance to CO_2_ (Sorrentino et al., 2016). However, there is no clear evidence in the literature explaining the seemingly systemic increase in foliar accumulation of macro‐ and micronutrients as a result of VPD increase, as found in this meta‐analysis. In this regard, a parsimonious explanation is that this may be the result of a concentration effect stemming from higher transpiration rates, which facilitate the transport of nutrients to the leaf (e.g., Cramer et al., 2008; Houshmandfar et al., 2018; Kunrath et al., 2020), coupled with VPD‐mediated decreases in leaf area.

In addition to these leaf‐level responses, our data also indicate that these changes will be accompanied by whole‐plant developmental alterations, leading to reductions in branching and leaf number, which further reduce the plant's evaporative surface, ultimately changing plant architecture (Figure 4a). While this outcome is consistent with predictions from the hydraulic corollary of Darcy's law (McDowell & Allen, 2015), the data compiled in Figure 7 indicate that non‐crop plants, on average, are more negatively impacted by VPD increase than crops, particularly for changes in leaf area, leaf number, and whole‐plant dry mass. This may be due to artificial selection by breeders, which tends to accumulate favorable alleles maximizing radiation interception by the canopy (i.e., leaf number and area).

Finally, the meta‐analysis confirms another prediction from Darcy's law, which is that adaptation to high VPD is likely to favor plants with shorter stature (Figure 4a; McDowell & Allen, 2015) and this tendency was further confirmed by a quantitative relationship across 45 species (Figure 5c). In this response, the height of annual plants was more negatively impacted compared to that of perennials (Figure 7d), a difference that could be related to the fact that plant height is determined by environmental conditions experienced incrementally over the years in perennial species compared to annual plants (Givnish et al., 2014). Because maximum plant height is proportional to the ratio of precipitation to pan evaporation (Givnish et al., 2014) and taller plants exhibit larger vulnerability to embolism (Olson et al., 2018), this response to VPD may help prevent severe embolisms from taking place. In this regard, considering the existing relationship between stem length and xylem vessel diameter (Olson et al., 2018), the correlation linking VPD and plant height (Figure 5c) may be valuable for predicting embolism vulnerability as a function of future VPD trends, provided that it is confirmed that extended exposure to high VPD reduces xylem vessel size and embolism risk.

Combined, these findings indicate that increases in VPD would trigger co‐ordinated leaf and whole‐plant developmental acclimations that—while acting to reduce water consumption—decrease plant primary productivity and, potentially, the ability of terrestrial ecosystems to act as carbon sinks. We speculate that such decreases in growth may further amplify negative VPD effects on plant water balance by exposing a higher proportion of unshaded soil to increasing evaporative demand (e.g., Duan et al., 2016), generating a feedback loop that may favor a faster buildup of soil moisture deficits, particularly for drought‐prone environments.

### 4.3 Increases in VPD negatively impact yield, likely through a combination of vegetative and reproductive effects

The meta‐analysis revealed strong effects of increased VPD on traits and physiological variables affecting reproductive development and yield, which outline a potential mechanism for yield decreases. Specifically, increases in VPD generally led to a lower number of flowers and a shorter time to anther opening (Figure 4b), indicating that yield decreases arise in part from alterations to processes underlying successful pollination and flower set. In the case of maize, this premise is consistent with the meta‐analysis of Lobell et al. (2014) who hypothesized that part of the VPD‐driven yield decreases found in the U.S. Midwest could be attributable to direct effects on pollen and grain set. This may be the result of changes in the anthesis‐silking interval, mediated by a decrease in pollen viability in response to VPD (Fonseca & Westgate, 2005) and in silk elongation rate under high VPD conditions (Turc et al., 2016). Such effects, which reflect specific sensitivities of reproductive tissues to VPD, add to those directly stemming from decreases in stomatal conductance and aboveground tissue growth rates as a result of VPD increase, which would reduce radiation interception and photoassimilate availability. More sparse evidence points to changes in fruit/seed quality, such as size and composition, but trends for these effects could not be identified with the current limited body of literature (Figure 4b).

Taken together, the findings compiled in this analysis converge to indicate that future increases in VPD may alter primary productivity through two main “meta‐mechanisms,” synthesized in Figure 8: (1) decreased photoassimilate availability for plant growth, leading to decreased phytomass, radiation interception and carbon allocation to aboveground tissues; and (2) particular sensitivities of reproductive organs to VPD increase, which hamper reproductive success, although this evidence is comparatively more limited. This framework provides strong biological support to recent yield and satellite‐derived productivity analyses linking historical VPD increases to decreases in crop and ecosystem productivity (Lobell et al., 2014; Yuan et al., 2019), and with predictions inferred from the hydraulic corollary of Darcy's law (McDowell & Allen, 2015).

### 4.4 Recommendations for future research

Unavoidably, the vast majority of the studies leveraged for this meta‐analysis were conducted under controlled environment conditions, given the need to examine VPD effects independently of other potentially confounding environmental variables. In addition, as in any meta‐analysis, caution should be given to inferences made on the basis of regressions of plant responses to environmental variables in which species are the sources of variation, particularly when species responses are nonlinear or dependent on contingent effects. However, the internal coherence of the emerging physiological framework and its consistency with global observations and predictions that were not part of the database makes it suitable to use as a basis to suggest the following research directions:

1. *Imposing realistic VPD regimes that are expected to occur in the locations of interest*. It is likely that future VPD regimes will not occur with the same intensity, duration, and timing across regions. Despite this, in the assembled body of literature, justifications of the target VPD values were missing in most records. In this regard, future investigations should identify the timing, intensity, and duration of the VPD treatments such that they are reasonable and realistic for the target locations. Furthermore, due to complex interactive effects between VPD and other variables, experimental designs should factor in other location‐specific environmental variables such as soil type, water availability regime, soil and air temperatures, irradiance, photoperiod, and atmospheric CO_2_ levels.
2. *Designing infrastructure enabling the imposition of specific VPD regimes*. It is critical that future research efforts focus on exposing plants to target VPD regimes as explained in recommendation (1). To this end, a two‐pronged approach, consisting of the use of (i) “high‐fidelity” growth chambers that are able to impose a highly specific VPD regime and (ii) field‐based infrastructure (e.g., Lihavainen et al., 2016; Tullus et al., 2012) to investigate more complex, larger scale and longer term outcomes, is needed.
3. *Diversifying plant types to be examined for VPD responses*. Figure 1 points to a strong bias in the literature toward studying dicots, forbs, and woody/perennial, non‐crop plants. More specifically, ferns, gymnosperms, grasses, and non‐perennial plants are much less studied. Furthermore, the assembled literature under‐investigated intra‐genotypic diversity in trait responses to VPD despite findings that cultivars and ecotypes exhibited significantly different trait responses to VPD (Aliniaeifard & van Meeteren, 2014; Devi et al., 2015; Rashid et al., 2018; Reymond et al., 2004). For efforts targeting the mitigation of negative VPD effects on crops or ecosystems, studies examining intra‐genotypic variability in such responses could represent a promising, untapped potential for mitigating the negative effects of rising VPD using crop breeding or ecological engineering.
4. *Expanding the study of VPD effects to a wider array of biological processes*. Our meta‐analysis, and particularly the data reported in Figures 3 and 4, indicates that more research is needed to examine the effects of VPD on three major under‐investigated groups of traits: (i) leaf internal anatomy, mineral (particularly N), carbon and hormonal status; (ii) shoot and root architectural traits; and (iii) reproductive development, yield, and fruit/seed composition. In the literature, this latter aspect was the least developed (Figure 4b), and future research could focus on separately examining male and female organ growth in response to VPD (e.g., Fonseca & Westgate, 2005; Turc et al., 2016). In this integrated effort, the most useful approaches will be those that simultaneously examine traits that are expressed at different organ, tissue, or organizational levels in order to characterize potential trait trade‐offs and enable an organismal‐level understanding of plant responses to VPD.
5. *Identifying quantitative relationships underlying complex plant responses to VPD and integrating them into crop, ecohydrological, and climate models*. Future mechanistic frameworks linking key physiological processes to changes in VPD alone or in interaction with other variables, as outlined above, should be developed such that they are easily integrated into larger scale, process‐based models. Such approaches will be key to better predicting critical outcomes such as primary productivity, crop yields or impacts on global water, carbon and nitrogen cycles, and also to identify and evaluate management options and candidate ideotypes that could be deployed to mitigate negative VPD effects.

## 5 CONCLUSIONS

Overall, we outline a general, integrated physiological framework that is consistent with the hydraulic corollary of Darcy's law along with quantitative relationships that provide insight into the complex and systemic effects of VPD on plant productivity. The effects of VPD on plant productivity are not only mediated by acclimation of gas exchange, but also by targeted developmental and metabolic programming that alters growth rates, anatomy, hormonal balance, architecture, and tissue biochemical composition. Furthermore, reproductive organs seem to exhibit specific sensitivities to VPD that are partially independent from VPD effects on gas exchange. Most of these changes are not taken into account in models investigating climate change effects on agro‐systems and ecosystems.

Our results point to the need for more integrative research efforts along the five main research areas identified in our recommendations, with support from various disciplines including ecophysiology, functional ecology, ecohydrology, crop physiology, plant breeding, crop modeling, and climate science. At the core of this multidisciplinary effort, more insight is needed into the mechanistic basis of these responses (synthesized in Figure 8) and the extent of their underlying interspecific and intraspecific variability.

## Supporting information

Supplementary Material

## Data Availability

Data including literature source, species/genotypic information, growth conditions, and VPD effect size for each examined response variable will be deposited in the Dryad Digital Repository.
