# Supplementary material for: Systemic effects of rising atmospheric vapor pressure deficit on plant physiology and productivity
Source: Glob Chang Biol. 2021 Mar 8;27(9):1704–20. doi: 10.1111/gcb.15548 (PMC8251766; doi:10.1111/gcb.15548)
Supplement: Supplementary file 1 — Supplementary Material [file GCB-27-1704-s001.docx]

**Supporting information Table S1**. List of the 112 species and 104 publications used in the meta-analysis. Taxonomic and classification information is from the Taxonomic Name Resolution Service (v4.0) and USDA-NRCS Plants databases.

| **Accepted name** | **Accepted author^A^** | **Accepted family** | **Group** | **Duration^B^** | **Growth habit^C^** | **Reference** |
| --- | --- | --- | --- | --- | --- | --- |
| *Aegiceras corniculatum* | (L.) Blanco | Primulaceae | Dicot | Perennial | Shrub/Tree | Ball and Farquhar (1984); Ball (1988) |
| *Aeschynanthus hybr.* | — | Gesneriaceae | Dicot | — | — | Schussler (1992) |
| *Ageratum houstonianum* | Mill. | Asteraceae | Dicot | Annual/Perennial | Forb/Herb | Krizek et al. (1971) |
| *Allium cepa* | L. | Amaryllidaceae | Monocot | Perennial | Forb/Herb | Hoffman and Rawlins (1971) |
| *Alstonia scholaris* | (L.) R. Br. | Apocynaceae | Dicot | — | — | Cunningham (2005); Cunningham (2006) |
| *Arabidopsis thaliana* | (L.) Heynh. | Brassicaceae | Dicot | Annual | Forb/Herb | Lake and Woodward (2008); Aliniaeifard et al. (2014); Arve et al. (2017) |
| *Arachis hypogaea* | L. | Fabaceae | Dicot | Annual/Perennial | Forb/Herb | Lee et al. (1972); Mortley et al. (2000) |
| *Argyrodendron trifoliolatum* | F.Muell. | Malvaceae | Dicot | Perennial | Tree | Cunningham (2005); Cunningham (2006) |
| *Atriplex spongiosa* | F.Muell. | Amaranthaceae | Dicot | — | — | Salim (1989) |
| *Avicennia marina subsp. australasica* | (Walp.) J.Everett | Acanthaceae | Dicot | Perennial | Shrub/Tree | Ball and Farquhar (1984); Ball (1988) |
| *Begonia × hiemalis Fotsch* | — | Begoniaceae | Dicot | — | — | Mortensen (1986); Gislerød et al. (1987); Gislerød and Mortensen (1990) |
| *Begonia cheimantha* | — | Begoniaceae | Dicot | — | — | Mortensen (2000) |
| *Begonia reniformis* | Dryand. | Begoniaceae | Dicot | — | — | Schussler (1992) |
| *Beta vulgaris* | L. | Amaranthaceae | Dicot | Annual/Biennial | Forb/Herb | Hoffman and Rawlins (1971); Ford and Thorne (1974); Milford and Lawlor (1975) |
| *Betula pendula* | Roth | Betulaceae | Dicot | Perennial | Tree | Sellin et al. (2013); Parts et al. (2013); Rosenvald et al. (2014); Sellin et al. (2015); Lihavainen et al. (2016a); Lihavainen et al. (2016b); Kupper et al. (2017); Lihavainen et al. (2017) |
| *Brachypodium sylvaticum* | (Huds.) P.Beauv. | Poaceae | Monocot | Perennial | Graminoid | Leuschner (2002) |
| *Brassica oleracea* | L. | Brassicaceae | Dicot | Perennial | Forb/Herb | Ford and Thorne (1974) |
| *Campanula isophylla* | Moretti | Campanulaceae | Dicot | Perennial | Forb/Herb | Mortensen (1986) |
| *Campanula trachelium* | L. | Campanulaceae | Dicot | Perennial | Forb/Herb | Leuschner (2002) |
| *Capsicum annuum* | L. | Solanaceae | Dicot | Annual/Perennial | Forb/Herb/Subshrub | Bakker (1991); Zabri and Burrage (1998) |
| *Castanospermum australe* | Cunn. and C. Fraser ex Hook. | Fabaceae | Dicot | Perennial | Tree | Cunningham (2005); Cunningham (2006) |
| *Chlorophytum comosum* | (Thunb.) Jacques | Asparagaceae | Monocot | Perennial | Forb/Herb | Mortensen and Gislerød (1990) |
| *Chrysanthemum indicum* | L. | Asteraceae | Dicot | — | — | Schussler (1992) |
| *Chrysanthemum morifolium* | Ramat | Asteraceae | Dicot | Perennial | Shrub/Subshrub | Mortensen (1986); Gislerød et al. (1987); Gislerød and Nelson (1989); Mortensen (2000) |
| *Cirsium arvense* | (L.) Scop. | Asteraceae | Dicot | Perennial | Forb/Herb | Hunter et al. (1985) |
| *Cissus alata* | Jacq. | Vitaceae | Dicot | Perennial | Vine | Mortensen and Gislerød (1990) |
| *Cleistogenes squarrosa* | (Trin. ex Ledeb.) Keng | Poaceae | Monocot | — | Graminoid | Gong et al. (2017) |
| *Codiaeum variegatum* | (L.) Rumph. ex A.Juss. | Euphorbiaceae | Dicot | Perennial | Shrub/Tree | Mortensen and Gislerød (1990) |
| *Cucumis melo* | L. | Cucurbitaceae | Dicot | Annual | Forb/Herb/Vine | An et al. (2002) |
| *Cucumis sativus* | L. | Cucurbitaceae | Dicot | Annual | Forb/Herb/Vine | Mortensen (1986); Bakker (1991); van de Sanden and Veen (1992); Shibuya et al. (2017); Shibuya et al. (2018) |
| *Dieffenbachia seguine* | (Jacq.) Schott | Araceae | Monocot | Perennial | Forb/Herb | Mortensen and Gislerød (1990) |
| *Digitalis purpurea* | L. | Plantaginaceae | Dicot | Biennial | Forb/Herb | Leuschner (2002) |
| *Doritaenopsis* | — | Orchidaceae | Monocot | — | — | Jeon et al. (2006); Lee et al. (2018) |
| *Dracaena fragrans* | (L.) Ker Gawl. | Asparagaceae | Monocot | Perennial | Shrub/Tree | Mortensen and Gislerød (1990) |
| *Epipremnum pinnatum* | (L.) Engl. | Araceae | Monocot | Perennial | Forb/Herb/Vine | Mortensen and Gislerød (1990) |
| *Eucryphia lucida* | (Labill.) Baill. | Cunoniaceae | Dicot | Perennial | Shrub/Tree | Cunningham (2005); Cunningham (2006) |
| *Euphorbia pulcherrima* | Willd. ex Klotzsch | Euphorbiaceae | Dicot | Perennial | Shrub/Tree | Mortensen (1986); Gislerød et al. (1987); Schussler (1992); Mortensen (2000) |
| *Fagus sylvatica* | L. | Fagaceae | Dicot | Perennial | Tree | Lendzion and Leuschner (2008) |
| *Fatsia japonica* | (Thunb.) Decne. & Planch. | Araliaceae | Dicot | — | — | Mortensen and Gislerød (1990) |
| *Festuca arundinacea* | Schreb. | Poaceae | Monocot | Perennial | Graminoid | Sinclair et al. (2007) |
| *Ficus benjamina* | L. | Moraceae | Dicot | Perennial | Shrub/Tree | Mortensen and Gislerød (1990) |
| *Ficus elastica* | Roxb. ex Hornem. | Moraceae | Dicot | Perennial | Tree | Mortensen and Gislerød (1990) |
| *Ficus pumila* | L. | Moraceae | Dicot | Perennial | Forb/Herb/Vine | Mortensen and Gislerød (1990) |
| *Geum urbanum* | L. | Rosaceae | Dicot | Perennial | Forb/Herb | Leuschner (2002) |
| *Glycine max* | (L.)Merr. | Fabaceae | Dicot | Annual | Forb/Herb | Woodward and Begg (1976); An et al. (2001); Roriz et al. (2014); Devi et al. (2015) |
| *Gossypium hirsutum* | L. | Malvaceae | Dicot | Annual/Perennial | Shrub/Subshrub/Tree | Hoffman et al. (1971) |
| *Hedera helix* | L. | Araliaceae | Dicot | Perennial | Vine | Mortensen and Gislerød (1990) |
| *Helianthus annuus* | L. | Asteraceae | Dicot | Annual | Forb/Herb | Salim (1989) |
| *Hieracium sylvaticum* | (L.) L. | Asteraceae | Dicot | — | — | Leuschner (2002) |
| *Hordelymus europaeus* | (L.) Jess. ex Harz | Poaceae | Monocot | Perennial | Graminoid | Leuschner (2002) |
| *Hordeum vulgare* | L. | Poaceae | Monocot | Annual | Graminoid | Hoffman and Jobes (1978) |
| *Hydrangea macrophylla* | (Thunb.) Ser. | Hydrangeaceae | Dicot | — | — | Codarin et al. (2006) |
| *Impatiens New Guinea hybr.* | — | Balsaminaceae | Dicot | Perennial | Forb/Herb | Schussler (1992) |
| *Ipomoea batatas* | (L.) Lam. | Convolvulaceae | Dicot | Annual/Perennial | Forb/Herb/Vine | Mortley et al. (1994) |
| *Jatropha curcas* | L. | Euphorbiaceae | Dicot | Perennial | Shrub/Tree | Rodrigues et al. (2016) |
| *Kalanchoe blossfeldiana* | Poelln. | Crassulaceae | Dicot | Perennial | Forb/Herb | Schussler (1992); Mortensen (2000) |
| *Lactuca sativa* | L. | Asteraceae | Dicot | Annual/Biennial/Perennial | Forb/Herb | Tibbitts and Bottenberg (1976); (Collier and Tibbitts, 1984); Mortensen (1986) |
| *Lupinus albus* | L. | Fabaceae | Dicot | Annual | Forb/Herb | Withers (1979) |
| *Malus pumila* | Mill. | Rosaceae | Dicot | Perennial | Tree | Tromp and Oele (1972) |
| *Maranta leuconeura* | E.Morren | Marantaceae | Monocot | — | — | Mortensen and Gislerød (1990) |
| *Medicago sativa* | L. | Fabaceae | Dicot | Annual/Perennial | Forb/Herb | De Luis et al. (2002) |
| *Mercurialis perennis* | L. | Euphorbiaceae | Dicot | Perennial | Forb/Herb | Lendzion and Leuschner (2009) |
| *Monstera deliciosa* | Liebm. | Araceae | Monocot | Perennial | Forb/Herb/Vine | Mortensen and Gislerød (1990) |
| *Nephrolepis cordifolia* | (L.) C. Presl | Davalliaceae | Fern | Perennial | Forb/Herb | Mortensen and Gislerød (1990) |
| *Nephrolepis exaltata* | (L.) Schott | Davalliaceae | Fern | Perennial | Forb/Herb | Mortensen (1986); Gislerød et al. (1987) |
| *Nothofagus cunninghamii* | (Hook.) Oerst. | Nothofagaceae | Dicot | Perennial | Tree | Cunningham (2005); Cunningham (2006); Hovenden et al. (2012) |
| *Oryza glaberrima* | Steud. | Poaceae | Monocot | Annual | Graminoid | Parent et al. (2010) |
| *Oryza sativa* | L. | Poaceae | Monocot | Annual | Graminoid | Parent et al. (2010) |
| *Panicum coloratum* | L. | Poaceae | Monocot | Perennial | Graminoid | Seneweera et al. (1998) |
| *Pellaea rotundifolia* | (G. Forst.) Hook. | Pteridaceae | Fern | Perennial | Forb/Herb | Mortensen and Gislerød (1990) |
| *Peperomia rotundifolia* | (L.) Kunth | Piperaceae | Dicot | Perennial | Forb/Herb/Vine | Mortensen and Gislerød (1990) |
| *Petunia hybrida* | Vilm. | Solanaceae | Dicot | Annual/Perennial | Forb/Herb | Krizek et al. (1971) |
| *Phalaenopsis* | — | Orchidaceae | Monocot | — | — | Cha-um et al. (2010); Kim et al. (2018); Lee et al. (2018) |
| *Phaseolus vulgaris* | L. | Fabaceae | Dicot | Annual | Forb/Herb/Vine | Salim (1989) |
| *Philodendron hederaceum* | (Jacq.) Schott | Araceae | Monocot | Perennial | Forb/Herb/Vine | Mortensen and Gislerød (1990) |
| *Picea glauca* | (Moench) Voss | Pinaceae | Gymnosperm | Perennial | Tree | Roberts and Zwiazek (2001) |
| *Picea mariana* | (Mill.) Britton, Sterns & Poggenb. | Pinaceae | Gymnosperm | Perennial | Tree | Darlington et al. (1997) |
| *Pinus banksiana* | Lamb. | Pinaceae | Gymnosperm | Perennial | Tree | Darlington et al. (1997) |
| *Populus nigra* | L. | Salicaceae | Dicot | Perennial | Tree | Rasheed et al. (2015) |
| *Populus tremula L. × P. tremuloides Michx.* | Michx. | Salicaceae | Dicot | Perennial | Tree | Tullus et al. (2012); Rosenvald et al. (2014); Jasińska et al. (2015); Lihavainen et al. (2016a) |
| *Prunus armeniaca* | L. | Rosaceae | Dicot | Perennial | Tree | Gradziel and Weinbaum (1999) |
| *Prunus dulcis* | (Mill.) D.A.Webb | Rosaceae | Dicot | Perennial | Tree | Gradziel and Weinbaum (1999) |
| *Prunus persica* | (L.) Batsch | Rosaceae | Dicot | Perennial | Tree | Gradziel and Weinbaum (1999) |
| *Radermachera sinica* | (Hance) Hemsl. | Bignoniaceae | Dicot | Perennial | Tree | Mortensen and Gislerød (1990) |
| *Raphanus raphanistrum subsp. sativus* | (L.) Domin | Brassicaceae | Dicot | Annual/Biennial | Forb/Herb | Hoffman and Rawlins (1971) |
| *Rhizophora apiculata* | Blume | Rhizophoraceae | Dicot | Perennial | Tree | Ball et al. (1997) |
| *Rhizophora stylosa* | Griff. | Rhizophoraceae | Dicot | Perennial | Tree | Ball et al. (1997) |
| *Rosa × hybrida* | — | Rosaceae | Dicot | Perennial | Shrub/Subshrub | Mortensen (1986); Mortensen et al. (2001); Torre et al. (2003); Fanourakis et al. (2011); Arve et al. (2013); Fanourakis et al. (2013); Giday et al. (2013) |
| *Rumex sanguineus* | L. | Polygonaceae | Dicot | Perennial | Forb/Herb | Leuschner (2002) |
| *Saintpaulia ionantha* | H.Wendl. | Gesneriaceae | Dicot | Perennial | Forb/Herb | Mortensen (1986); Gislerød et al. (1987); Schussler (1992) |
| *Saxifraga stolonifera* | Curtis | Saxifragaceae | Dicot | Perennial | Forb/Herb | Mortensen and Gislerød (1990) |
| *Schefflera actinophylla* | (Endl.) Harms | Araliaceae | Dicot | Perennial | Tree | Mortensen and Gislerød (1990) |
| *Schefflera arboricola* | (Hayata) Merr. | Araliaceae | Dicot | Perennial | Shrub/Tree | Mortensen and Gislerød (1990) |
| *Scrophularia nodosa* | L. | Scrophulariaceae | Dicot | Perennial | Forb/Herb | Leuschner (2002) |
| *Sloanea woollsii F. Muell. (Elaeocarpaceae)* | — | Elaeocarpaceae | Dicot | Perennial | Tree | Cunningham (2005); Cunningham (2006) |
| *Solanum lycopersicum* | Mill. | Solanaceae | Dicot | Annual/Perennial | Forb/Herb | Armstrong and Kirkby (1979); Mortensen (1986); Gislerød et al. (1987); Salim (1989); Bakker (1991); Schussler (1992); Lopez et al. (1998); Gautier et al. (1999); Bertin (2000); Mulholland et al. (2000); Mulholland et al. (2001); Lorenzo et al. (2003); An et al. (2005); Lu et al. (2015); Zhang et al. (2015); Arve et al. (2017); Zhang et al. (2017); Du et al. (2018) |
| *Solanum tuberosum* | L. | Solanaceae | Dicot | Perennial | Forb/Herb/Subshrub | Wheeler et al. (1989) |
| *Soleirolia soleirolii* | (Req.) Dandy | Urticaceae | Dicot | Perennial | Forb/Herb | Mortensen (1986) |
| *Spinacia Oleracera* | L. | Amaranthaceae | Dicot | Annual | Forb/Herb | Iwabuchi et al. (1996) |
| *Stachys sylvatica* | L. | Lamiaceae | Dicot | Perennial | Forb/Herb | Lendzion and Leuschner (2009) |
| *Syngonium podophyllum* | Schott | Araceae | Monocot | Perennial | Vine | Mortensen and Gislerød (1990) |
| *Syzygium smithii* | (Poir.) Nied. | Myrtaceae | Dicot | Perennial | Tree | Cunningham (2005); Cunningham (2006) |
| *Tagetes erecta* | L. | Asteraceae | Dicot | Annual | Forb/Herb | Krizek et al. (1971) |
| *Theobroma cacao* | L. | Malvaceae | Dicot | Perennial | Tree | Salef (1970) |
| *Toona ciliata* | M.Roem. | Meliaceae | Dicot | Perennial | Tree | Carins Murphy et al. (2014) |
| *Tradescantia virginiana* | L. | Commelinaceae | Monocot | Perennial | Forb/Herb | Rezaei Nejad and van Meeteren (2005); Rezaei Nejad et al. (2006); Rezaei Nejad and van Meeteren (2007); Rezaei Nejad and van Meeteren (2008) |
| *Tristaniopsis laurina* | (Sm.) Peter G.Wilson & J.T.Waterh. | Myrtaceae | Dicot | Perennial | Tree | Cunningham (2005); Cunningham (2006) |
| *Triticum aestivum* | L. | Poaceae | Monocot | Annual | Graminoid | Ford and Thorne (1974); Hoffman and Jobes (1978); Rashid et al. (2018) |
| *Vicia faba* | L. | Fabaceae | Dicot | Annual | Forb/Herb/Vine | Aliniaeifard et al. (2014) |
| *Vigna radiata* | (L.) R.Wilczek | Fabaceae | Dicot | Annual | Forb/Herb | Salim (1989) |
| *Yucca aloifolia* | L. | Asparagaceae | Monocot | Perennial | Shrub/Tree | Mortensen and Gislerød (1990) |
| *Zea mays* | L. | Poaceae | Monocot | Annual | Graminoid | Hoffman and Jobes (1978); Ben Haj Salah and Tardieu (1996); Ben Haj Salah and Tardieu (1997); Reymond et al. (2003); Reymond et al. (2004); Sadok et al. (2007); Welcker et al. (2011); Turc et al. (2016) |

^A^—: no author name was identified in the database or database returned “No opinion” regarding the taxonomic status.

^B,C^—: unavailable information.


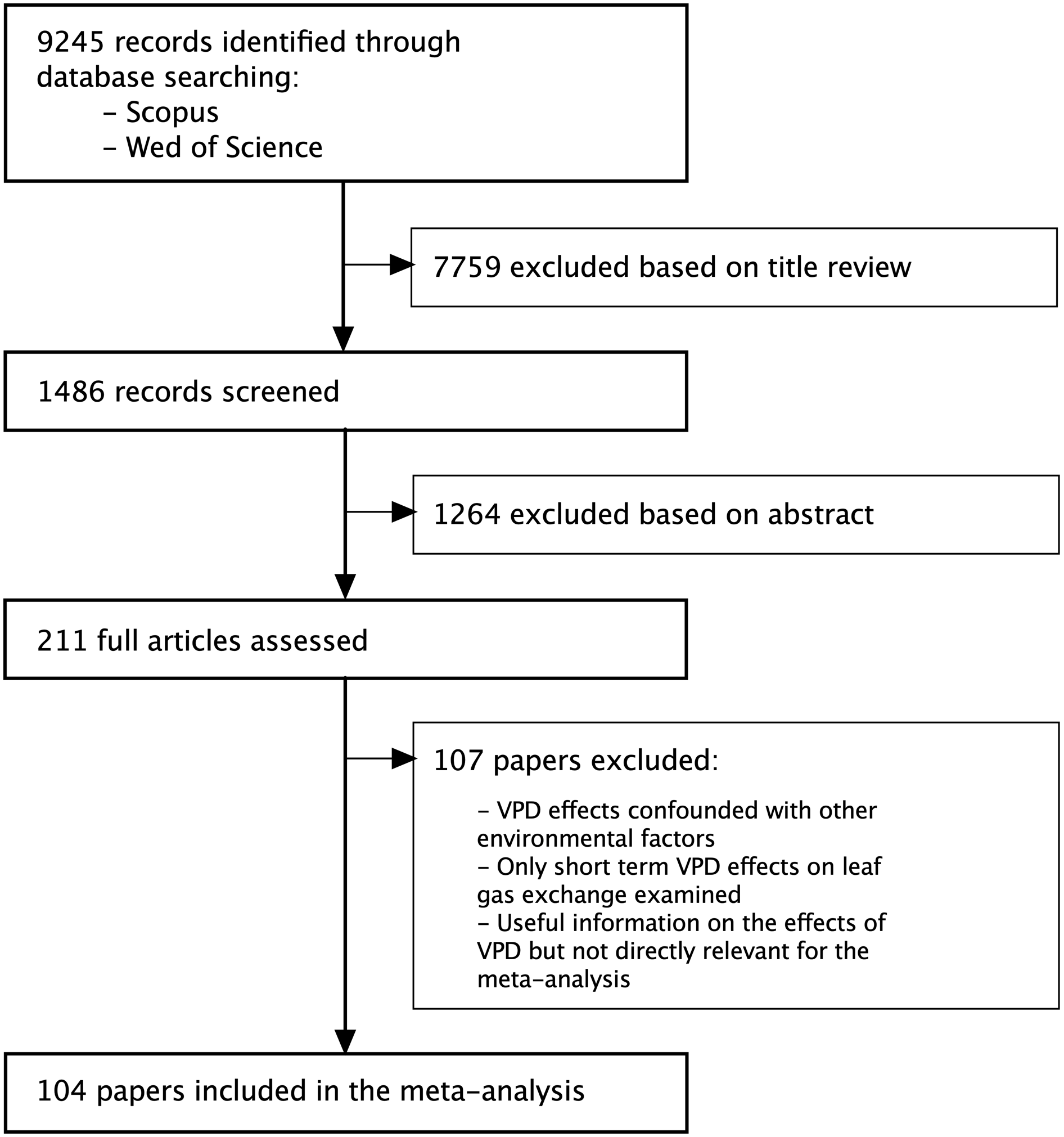


**Figure S1**. General procedure followed for identified the records used the in the meta-analysis.

**References**

Aliniaeifard, S., Malcolm Matamoros, P., & van Meeteren, U. (2014). Stomatal malfunctioning under low VPD conditions: induced by alterations in stomatal morphology and leaf anatomy or in the ABA signaling. *Physiologia Plantarum*, *152*(4), 688-699. doi:10.1111/ppl.12216

An, P., Inanaga, S., Kafkafi, U., Lux, A., & Sugimoto, Y. (2001). Different effect of humidity on growth and salt tolerance of two soybean cultivars. *Biologia Plantarum*, *44*(3), 405-410.

An, P., Inanaga, S., Lux, A., Li, X. J., Ali, M. E. K., Matsui, T., & Sugimoto, Y. (2002). Effects of salinity and relative humidity on two melon cultivars differing in salt tolerance. *Biologia Plantarum*, *45*(3), 409-415.

An, P., Inanaga, S., Li, X. J., Eneji, A. E., & Zhu, N. W. (2005). Interactive Effects of Salinity and Air Humidity on Two Tomato Cultivars Differing in Salt Tolerance. *Journal of Plant Nutrition*, *28*(3), 459-473. doi:10.1081/pln-200049177

Armstrong, M. J., & Kirkby, A. (1979). The influence of humidity on the mineral composition of tomato plants with special reference to calcium distribution. *Plant and Soil*, *52*(3), 427-435.

Arve, L. E., Terfa, M. T., Gislerød, H. R., Olsen, J. E., & Torre, S. (2013). High relative air humidity and continuous light reduce stomata functionality by affecting the ABA regulation in rose leaves. *Plant, Cell & Environment*, *36*(2), 382-392. doi:10.1111/j.1365-3040.2012.02580.x

Arve, L. E., Kruse, O. M. O., Tanino, K. K., Olsen, J. E., Futsæther, C., & Torre, S. (2017). Daily changes in VPD during leaf development in high air humidity increase the stomatal responsiveness to darkness and dry air. *Journal of Plant Physiology*, *211*, 63-69. doi:10.1016/j.jplph.2016.12.011

Bakker, J. C. (1991). Effects of humidity on stomatal density and its relation to leaf conductance. *Scientia Horticulturae*, *48*, 205-212.

Ball, M. C. (1988). Salinity tolerance in the mangroves Aegiceras corniculatum and Avicennia marina I. Water use in relation to growth, carbon partitioning, and salt tolerance. *Australian Journal of Plant Physiology*, *15*, 447-464.

Ball, M. C., Cochrane, M. J., & Rawson, H. M. (1997). Growth and water use of the mangroves Rhizophora apiculata and R. stylosa in response to salinity and humidity under ambient and elevated concentrations of atmospheric CO2. *Plant, Cell & Environment*, *20*, 1158-1166.

Ball, M. C., & Farquhar, G. D. (1984). Photosynthetic and Stomatal Responses of Two Mangrove Species, Aegiceras corniculatum and Avicennia marina, to Long Term Salinity and Humidity Conditions. *Plant Physiology*, *74*(1), 1-6. doi:10.2307/4268397

Ben Haj Salah, H., & Tardieu, F. (1996). Quantitative analysis of the combined effects of temperature, evaporative demand and light on leaf elongation rate in well-wateredfieldf and laboratory-grown maize plants. *Journal of Experimental Botany*, *47*(304), 1689-1698.

Ben Haj Salah, H., & Tardieu, F. (1997). Control of leaf expansion rate of droughted maize plants under fluctuating evaporative demand. *Plant Physiology*, *114*, 893-900.

Bertin, N. (2000). Seasonal Evolution of the Quality of Fresh Glasshouse Tomatoes under Mediterranean Conditions, as Affected by Air Vapour Pressure Deficit and Plant Fruit Load. *Annals of Botany*, *85*(6), 741-750. doi:10.1006/anbo.2000.1123

Bianchini, M., & Pacini, E. (1996). Explosive Anther Dehiscence in Ricinus communis L. Involves Cell Wall Modifications and Relative Humidity. *International Journal of Plant Sciences*, *157*(6), 739-745. doi:10.2307/2474887

Carins Murphy, M. R., Jordan, G. J., & Brodribb, T. J. (2014). Acclimation to humidity modifies the link between leaf size and the density of veins and stomata. *Plant, Cell & Environment*, *37*(1), 124-131. doi:10.1111/pce.12136

Cha-um, S., Ulziibat, B., & Kirdmanee, C. (2010). Effects of temperature and relative humidity during in vitro acclimatization, on physiological changes and growth characters of Phalaenopsis adapted to in vivo. *Australian Journal of Crop Science*, *4*(9), 750-756.

Codarin, S., Galopin, G., & Chasseriaux, G. (2006). Effect of air humidity on the growth and morphology of Hydrangea macrophylla L. *Scientia Horticulturae*, *108*(3), 303-309. doi:10.1016/j.scienta.2006.01.036

Collier, G. F., & Tibbitts, T. W. (1984). Effects of relative humidity and root temperature on calcium concentration and tipburn development in lettuce. *Journal of the American Society for Horticultural Science*, *109*(2), 128-131.

Cunningham, S. C. (2005). Photosynthetic responses to vapour pressure deficit in temperate and tropical evergreen rainforest trees of Australia. *Oecologia*, *142*(4), 521-528. doi:10.1007/s00442-004-1766-1

Cunningham, S. C. (2006). Effects of vapour pressure deficit on growth of temperate and tropical evergreen rainforest trees of Australia. *Acta Oecologica*, *30*(3), 399-406. doi:10.1016/j.actao.2006.05.009

Darlington, A. B., Halinska, A., Dat, J. F., & Blake, T. J. (1997). Effects of increasing saturation vapour pressure deficit on growth and ABA levels in black spruce and jack pine. *Trees - Structure and Function*, *11*(4), 223-228.

De Luis, I., Irigoyen, J. J., & Sánchez-Díaz, M. (2002). Low vapour pressure deficit reduces the beneficial effect of elevated CO2 on growth of N2 -fixing alfalfa plants. *Physiologia Plantarum*, *116*(4), 497-502.

Devi, M. J., Taliercio, E. W., & Sinclair, T. R. (2015). Leaf expansion of soybean subjected to high and low atmospheric vapour pressure deficits. *Journal of Experimental Botany*, *66*(7), 1845-1850. doi:10.1093/jxb/eru520

Du, Q., Zhang, D., Jiao, X., Song, X., & Li, J. (2018). Effects of atmospheric and soil water status on photosynthesis and growth in tomato. *Plant, Soil and Environment*, *64*(No. 1), 13-19. doi:10.17221/701/2017-pse

Fanourakis, D., Carvalho, S. M. P., Almeida, D. P. F., & Heuvelink, E. (2011). Avoiding high relative air humidity during critical stages of leaf ontogeny is decisive for stomatal functioning. *Physiologia Plantarum*, *142*(3), 274-286. doi:10.1111/j.1399-3054.2011.01475.x

Fanourakis, D., Heuvelink, E., & Carvalho, S. M. P. (2013). A comprehensive analysis of the physiological and anatomical components involved in higher water loss rates after leaf development at high humidity. *Journal of Plant Physiology*, *170*(10), 890-898. doi:10.1016/j.jplph.2013.01.013

Ford, M. A., & Thorne, G. N. (1974). Effects of atmospheric humidity on plant growth. *Annals of Botany*, *38*(2), 441-452.

Gautier, H., Tchamitchian, M., & S, G. (1999). Effects of decreasing VPD by misting on leaf area and leaf inclination in tomato and estimation of consequences on light absorption. *Acta Horticulturae*, *507*, 77-84.

Giday, H., Fanourakis, D., Kjaer, K. H., Fomsgaard, I. S., & Ottosen, C. O. (2013). Foliar abscisic acid content underlies genotypic variation in stomatal responsiveness after growth at high relative air humidity. *Annals of Botany*, *112*(9), 1857-1867. doi:10.1093/aob/mct220

Gislerød, H. R., & Mortensen, L. M. (1990). Relative humidity and nutrient concentration affect nutrient uptake and growth of Begonia × hiemalis. *HortScience*, *25*(5), 524-526.

Gislerød, H. R., & Nelson, P. V. (1989). The interaction of relative air humidity and carbon dioxide enrichment in the growth of Chrysanthemum × morifolium Ramat. *Scientia Horticulturae*, *38*(3-4), 305-313.

Gislerød, H. R., Selmer-Olsen, A. R., & Mortensen, L. M. (1987). The effect of air humidity on nutrient uptake of some greenhouse plants. *Plant and Soil*, *102*(2), 193-196.

Gong, X. Y., Schäufele, R., & Schnyder, H. (2017). Bundle-sheath leakiness and intrinsic water use efficiency of a perennial C4 grass are increased at high vapour pressure deficit during growth. *Journal of Experimental Botany*, *68*(2), 321-333. doi:10.1093/jxb/erw417

Gradziel, T. M., & Weinbaum, S. A. (1999). High relative humidity reduces anther dehiscence in apricot, peach, and almond. *HortScience*, *34*(2), 322-325.

Hoffman, G. J., & Jobes, J. A. (1978). Growth and water relations of cereal crops as influenced by salinity and relative humidity. *Agronomy Journal*, *70*, 765-769.

Hoffman, G. J., & Rawlins, S. L. (1971). Growth and water potential of root crops as influenced by salinity and relative humidity. *Agronomy Journal*, *63*, 877-880.

Hoffman, G. J., Rawlins, S. L., Garber, M. J., & Cullen, E. M. (1971). Water relations and growth of cotton as influenced by salinity and relative humidity. *Agronomy Journal*, *63*, 822-826.

Hovenden, M. J., Vander Schoor, J. K., & Osanai, Y. (2012). Relative humidity has dramatic impacts on leaf morphology but little effect on stomatal index or density in Nothofagus cunninghamii (Nothofagaceae). *Australian Journal of Botany*, *60*(8), 700. doi:10.1071/bt12110

Hunter, J. H., Hsiao, A. I., & McIntyre, G. I. (1985). Some Effects of Humidity on the Growth and Development of Cirsium arvense. *Botanical Gazette*, *146*(4), 483-488. doi:10.2307/2474625

Iwabuchi, K., Saito, G., Goto, E., & Takakura, T. (1996). Effect of vapor pressure deficit on spinach growth under hypobaric conditions. *Acta Horticulturae*, *440*, 60-64.

Jasińska, A. K., Alber, M., Tullus, A., Rahi, M., & Sellin, A. (2015). Impact of elevated atmospheric humidity on anatomical and hydraulic traits of xylem in hybrid aspen. *Functional Plant Biology*, *42*(6), 565. doi:10.1071/fp14224

Jeon, M.-W., Ali, M. B., Hahn, E.-J., & Paek, K.-Y. (2006). Photosynthetic pigments, morphology and leaf gas exchange during ex vitro acclimatization of micropropagated CAM Doritaenopsis plantlets under relative humidity and air temperature. *Environmental and Experimental Botany*, *55*(1-2), 183-194. doi:10.1016/j.envexpbot.2004.10.014

Kim, J. K., Yoon, Y. J., Kim, K. S., Na, J.-K., & Choi, K. Y. (2018). Effects of relative humidity and air injection on physiological and stomatal responses in phalaenopsis during acclimatization. *Horticultural Science and Technology*, *36*(2), 193-201.

Krizek, D. T., Bailey, W. A., & Klueter, H. H. (1971). Effects of relative humidity and type of container on the growth of F1 hybrid annuals in controlled environments. *American Journal of Botany*, *58*(6), 544-551.

Kupper, P., Rohula, G., Inno, L., Ostonen, I., Sellin, A., & Sõber, A. (2017). Impact of high daytime air humidity on nutrient uptake and night-time water flux in silver birch, a boreal forest tree species. *Regional Environmental Change*, *17*(7), 2149-2157. doi:10.1007/s10113-016-1092-2

Lake, J. A., & Woodward, F. I. (2008). Response of stomatal numbers to CO2 and humidity: control by transpiration rate and abscisic acid. *New Phytologist*, *179*(2), 397-404. doi:10.1111/j.1469-8137.2008.02485.x

Lee, H. B., Lim, S. H., Lim, N. H., An, S. K., & Kim, K. S. (2018). Growth and CO2 exchange in young Phalaenopsis orchids grown under different levels of humidity during the vegetative period. *Horticulture, Environment, and Biotechnology*, *59*(1), 37-43. doi:10.1007/s13580-018-0005-3

Lee, T. A., Jr., Ketring, D. L., & Powell, R. D. (1972). Flowering and Growth Response of Peanut Plants (Arachis hypogaea L. var. Starr) at Two Levels of Relative Humidity. *Plant Physiology*, *49*(2), 190-193. doi:10.2307/4262689

Lendzion, J., & Leuschner, C. (2008). Growth of European beech (Fagus sylvatica L.) saplings is limited by elevated atmospheric vapour pressure deficits. *Forest Ecology and Management*, *256*(4), 648-655. doi:10.1016/j.foreco.2008.05.008

Lendzion, J., & Leuschner, C. (2009). Temperate forest herbs are adapted to high air humidity — evidence from climate chamber and humidity manipulation experiments in the field. *Canadian Journal of Forest Research*, *39*(12), 2332-2342. doi:10.1139/x09-143

Leuschner, C. (2002). Air humidity as an ecological factor for woodland herbs: leaf water status, nutrient uptake, leaf anatomy, and productivity of eight species grown at low or high vpd levels. *Flora*, *197*(4), 262-274.

Lihavainen, J., Keinänen, M., Keski-Saari, S., Kontunen-Soppela, S., Sõber, A., & Oksanen, E. (2016a). Artificially decreased vapour pressure deficit in field conditions modifies foliar metabolite profiles in birch and aspen. *Journal of Experimental Botany*, *67*(14), 4367-4378. doi:10.1093/jxb/erw219

Lihavainen, J., Ahonen, V., Keski-Saari, S., Kontunen-Soppela, S., Oksanen, E., & Keinänen, M. (2016b). Low vapour pressure deficit affects nitrogen nutrition and foliar metabolites in silver birch. *Journal of Experimental Botany*, *67*(14), 4353-4365. doi:10.1093/jxb/erw218

Lihavainen, J., Ahonen, V., Keski-Saari, S., Sõber, A., Oksanen, E., & Keinänen, M. (2017). Low vapor pressure deficit reduces glandular trichome density and modifies the chemical composition of cuticular waxes in silver birch leaves. *Tree Physiology*, *37*(9), 1166-1181. doi:10.1093/treephys/tpx045

Lopez, J., Dorais, M., Tremblay, N., & Gosselin, A. (1998). Effects ok varying sulfate concentrations and vapor pressure deficits (VPD) on greenhouse tomato fruit quality, foliar nutrient concentration and amino acid components. *Acta Horticulturae*, *458*, 303-310.

Lorenzo, P., Sánchez-Guerrero, M. C., & Medrano, E. (2003). Effect of vapour pressure deficit on growth, development and dry matter allocation of tomato plants. *Acta Horticulturae*, *614*, 863-867.

Lu, N., Nukaya, T., Kamimura, T., Zhang, D., Kurimoto, I., Takagaki, M., . . . Yamori, W. (2015). Control of vapor pressure deficit (VPD) in greenhouse enhanced tomato growth and productivity during the winter season. *Scientia Horticulturae*, *197*, 17-23. doi:10.1016/j.scienta.2015.11.001

Milford, G. F. J., & Lawlor, D. W. (1975). Effects of varying air and soil moisture on the water relations and growth of sugar beet. *Annals of Applied Biology*, *80*(1), 93-102.

Mortensen, L. M. (1986). Effect of relative humidity on growth and flowering of some greenhouse plants. *Scientia Horticulturae*, *29*(4), 301-307.

Mortensen, L. M. (2000). Effects of air humidity on growth, flowering, keeping quality and water relations of four short-day greenhouse species. *Scientia Horticulturae*, *86*(4), 299-310.

Mortensen, L. M., & Gislerød, H. R. (1990). Effects of air humidity and supplementary lighting on foliage plants. *Scientia Horticulturae*, *44*(3-4), 301-308.

Mortensen, L. M., Ottosen, C.-O., & Gislerød, H. R. (2001). Effects of air humidity and K:Ca ratio on growth, morphology, flowering and keeping quality of pot roses. *Scientia Horticulturae*, *90*(1-2), 131-141.

Mortley, D. G., Bonsi, C. K., Loretan, P. A., Hill, W. A., & Morris, C. E. (1994). Relative humidity influences yield, edible biomass, and linear growth rate of sweetpotato. *HortScience*, *29*(6), 609-610.

Mortley, D. G., Bonsi, C. K., Loretan, P. A., Hill, W. A., & Morris, C. E. (2000). High relative humidity increases yield, harvest index, flowering, and gynophore growth of hydroponically grown peanut plants. *HortScience*, *35*(1), 46-48.

Mulholland, B. J., Fussell, M., Edmondson, R. N., Basham, J., & McKee, J. M. T. (2001). Effect of vpd, K nutrition and root-zone temperature on leaf area development, accumulation of Ca and K and yield in tomato. *Journal of Horticultural Science and Biotechnology*, *76*(5), 641-647.

Mulholland, B. J., Fussell, M., Edmondson, R. N., Burns, I. G., McKee, J. M. T., & Basham, J. (2000). Effect of humidity and nutrient feed K/Ca ratio on physiological responses and the accumulation of dry matter, Ca and K in tomato. *Journal of Horticultural Science and Biotechnology*, *75*(6), 713-722. doi:10.1080/14620316.2000.11511312

Parent, B., Suard, B., Serraj, R., & Tardieu, F. (2010). Rice leaf growth and water potential are resilient to evaporative demand and soil water deficit once the effects of root system are neutralized. *Plant, Cell & Environment*, *33*(8), 1256-1267. doi:10.1111/j.1365-3040.2010.02145.x

Parts, K., Tedersoo, L., Lõhmus, K., Kupper, P., Rosenvald, K., Sõber, A., & Ostonen, I. (2013). Increased air humidity and understory composition shape short root traits and the colonizing ectomycorrhizal fungal community in silver birch stands. *Forest Ecology and Management*, *310*, 720-728. doi:10.1016/j.foreco.2013.09.017

Rasheed, F., Dreyer, E., Richard, B., Brignolas, F., Brendel, O., & Le Thiec, D. (2015). Vapour pressure deficit during growth has little impact on genotypic differences of transpiration efficiency at leaf and whole-plant level: an example from Populus nigra L. *Plant, Cell & Environment*, *38*(4), 670-684. doi:10.1111/pce.12423

Rashid, M. A., Andersen, M. N., Wollenweber, B., Zhang, X., & Olesen, J. E. (2018). Acclimation to higher VPD and temperature minimized negative effects on assimilation and grain yield of wheat. *Agricultural and Forest Meteorology*, *248*, 119-129. doi:10.1016/j.agrformet.2017.09.018

Reymond, M., Muller, B., Leonardi, A., Charcosset, A., & Tardieu, F. (2003). Combining quantitative trait loci analysis and an ecophysiological model to analyze the genetic variability of the responses of maize leaf growth to temperature and water deficit. *Plant Physiology*, *131*(2), 664-675. doi:10.1104/pp.013839

Reymond, M., Muller, B., & Tardieu, F. (2004). Dealing with the genotypexenvironment interaction via a modelling approach: a comparison of QTLs of maize leaf length or width with QTLs of model parameters. *Journal of Experimental Botany*, *55*(407), 2461-2472. doi:10.1093/jxb/erh200

Rezaei Nejad, A., Harbinson, J., & van Meeteren, U. (2006). Dynamics of spatial heterogeneity of stomatal closure in Tradescantia virginiana altered by growth at high relative air humidity. *Journal of Experimental Botany*, *57*(14), 3669-3678. doi:10.1093/jxb/erl114

Rezaei Nejad, A., & van Meeteren, U. (2007). The role of abscisic acid in disturbed stomatal response characteristics of Tradescantia virginiana during growth at high relative air humidity. *Journal of Experimental Botany*, *58*(3), 627-636. doi:10.1093/jxb/erl234

Rezaei Nejad, A., & van Meeteren, U. (2008). Dynamics of adaptation of stomatal behaviour to moderate or high relative air humidity in Tradescantia virginiana. *Journal of Experimental Botany*, *59*(2), 289-301. doi:10.1093/jxb/erm308

Rezaei Nejad, A., & van Meeteren, U. (2005). Stomatal response characteristics of Tradescantia virginiana grown at high relative air humidity. *Physiologia Plantarum*, *125*(3), 324-332. doi:10.1111/j.1399-3054.2005.00567.x

Roberts, J. J., & Zwiazek, J. J. (2001). Growth, morphology, and gas exchange in white spruce (<i>Picea glauca</i>) seedlings acclimated to different humidity conditions. *Canadian Journal of Forest Research*, *31*(6), 1038-1045. doi:10.1139/cjfr-31-6-1038

Rodrigues, C. R. F., Silveira, J. A. G., Viégas, R. A., Moura, R. M., Aragão, R. M., & Silva, E. N. (2016). Combined effects of high relative humidity and K + supply mitigates damage caused by salt stress on growth, photosynthesis and ion homeostasis in J. curcas plants. *Agricultural Water Management*, *163*, 255-262. doi:10.1016/j.agwat.2015.09.027

Roriz, M., Carvalho, S. M. P., & Vasconcelos, M. W. (2014). High relative air humidity influences mineral accumulation and growth in iron deficient soybean plants. *Frontiers in Plant Science*, *5*. doi:10.3389/fpls.2014.00726

Rosenvald, K., Tullus, A., Ostonen, I., Uri, V., Kupper, P., Aosaar, J., . . . Lõhmus, K. (2014). The effect of elevated air humidity on young silver birch and hybrid aspen biomass allocation and accumulation – Acclimation mechanisms and capacity. *Forest Ecology and Management*, *330*, 252-260. doi:10.1016/j.foreco.2014.07.016

Sadok, W., Naudin, P., Boussuge, B., Muller, B., Welcker, C., & Tardieu, F. (2007). Leaf growth rate per unit thermal time follows QTL-dependent daily patterns in hundreds of maize lines under naturally fluctuating conditions. *Plant, Cell & Environment*, *30*(2), 135-146. doi:10.1111/j.1365-3040.2006.01611.x

Salef, P. J. M. (1970). Growth and flowering of cacao under controlled atmospheric relative humidities. *Journal of Horticultural Science*, *45*(2), 119-132. doi:10.1080/00221589.1970.11514338

Salim, M. (1989). Effects of salinity and relative humidity on growth and ionic relations of plants. *New Phytologist*, *113*(1), 13-20. doi:10.2307/2557032

Schussler, H. K. (1992). The influence of different constant and fluctuating water vapour pressure gradients on morphogenesis. *Acta Horticulturae*, *327*, 105-110.

Sellin, A., Rosenvald, K., Õunapuu-Pikas, E., Tullus, A., Ostonen, I., & Lõhmus, K. (2015). Elevated air humidity affects hydraulic traits and tree size but not biomass allocation in young silver birches (Betula pendula). *Frontiers in Plant Science*, *6*, 860. doi:10.3389/fpls.2015.00860

Sellin, A., Tullus, A., Niglas, A., Õunapuu, E., Karusion, A., & Lõhmus, K. (2013). Humidity-driven changes in growth rate, photosynthetic capacity, hydraulic properties and other functional traits in silver birch (Betula pendula). *Ecological Research*, *28*(3), 523-535. doi:10.1007/s11284-013-1041-1

Seneweera, S. P., Ghannoum, O., & Conroy, J. (1998). High vapour pressure deficit and low soil water availability enhance shoot growth responses of a C4 grass (Panicum coloratum cv. Bambatsi) to CO2 enrichment. *Australian Journal of Plant Physiology*, *25*(3), 287-292.

Shibuya, T., Kano, K., Endo, R., & Kitaya, Y. (2018). Effects of the interaction between vapor-pressure deficit and salinity on growth and photosynthesis of Cucumis sativus seedlings under different CO2 concentrations. *Photosynthetica*, *56*(3), 893-900. doi:10.1007/s11099-017-0746-8

Shibuya, T., Kano, K., Endo, R., & Kitaya, Y. (2017). Photosynthetic properties and response to drought in cucumber seedlings acclimatized to different vapor-pressure-deficit levels. *Horticulture Journal*, *86*(3), 334-339. doi:10.2503/hortj.mi-154

Sinclair, T., Fiscus, E., Wherley, B., Durham, M., & Rufty, T. (2007). Atmospheric vapor pressure deficit is critical in predicting growth response of “cool-season” grass Festuca arundinacea to temperature change. *Planta*, *227*(1), 273-276. doi:10.1007/s00425-007-0645-5

Tibbitts, T. W., & Bottenberg, G. (1976). Growth of lettuce under controlled humidity levels. *Journal of the American Society for Horticultural Science*, *101*(1), 70-73.

Torre, S., Fjeld, T., Gislerød, H. R., & Moe, R. (2003). Leaf anatomy and stomatal morphology of greenhouse roses grown at moderate or high air humidity. *Journal of the American Society for Horticultural Science*, *128*(4), 598-602.

Tromp, J., & Oele, J. (1972). Shoot growth and mineral composition of leaves and fruits of apple as affected by relative air humidity. *Physiologia Plantarum*, *27*(2), 253-258.

Tullus, A., Kupper, P., Sellin, A., Parts, L., Sõber, J., Tullus, T., . . . Tullus, H. (2012). Climate change at northern latitudes: rising atmospheric humidity decreases transpiration, N-uptake and growth rate of hybrid aspen. *PLOS ONE*, *7*(8), e42648. doi:10.1371/journal.pone.0042648

Turc, O., Bouteillé, M., Fuad-Hassan, A., Welcker, C., & Tardieu, F. (2016). The growth of vegetative and reproductive structures (leaves and silks) respond similarly to hydraulic cues in maize. *New Phytologist*, *212*(2), 377-388. doi:10.1111/nph.14053

van de Sanden, P. A. C. M., & Veen, B. W. (1992). Effects of air humidity and nutrient solution concentration on growth, water potential and stomatal conductance of cucumber seedlings. *Scientia Horticulturae*, *50*(3), 173-186.

Welcker, C., Sadok, W., Dignat, G., Renault, M., Salvi, S., Charcosset, A., & Tardieu, F. (2011). A Common genetic determinism for sensitivities to soil water deficit and evaporative demand: meta-Analysis of quantitative trait loci and introgression lines of maize. *Plant Physiology*, *157*(2), 718-729. doi:10.1104/pp.111.176479

Wheeler, R. M., Tibbitts, T. W., & Fitzpatrick, A. H. (1989). Potato growth in response to relative humidity. *HortScience*, *24*(3), 482-484.

Withers, N. J. (1979). Effects of water stress on Lupinus albus. *New Zealand Journal of Agricultural Research*, *22*(3), 445-454. doi:10.1080/00288233.1979.10430773

Woodward, R. G., & Begg, J. E. (1976). The effect of atmospheric humidity on the yield and quality of soya bean. *Australian Journal of Agricultural Research*, *27*, 501-508.

Zabri, A. W., & Burrage, S. W. (1998). The effects of vapour pressure deficit (VPD) and enrichment with CO2 on photosynthesis, stomatal conductance, transpiration rate and water use efficiency (WUE) of sweet pepper (Capsicum annuum L.) grown by NFT. *Acta Horticulturae*, *458*, 351-356.

Zhang, D., Du, Q., Zhang, Z., Jiao, X., Song, X., & Li, J. (2017). Vapour pressure deficit control in relation to water transport and water productivity in greenhouse tomato production during summer. *Scientific Reports*, *7*, 43461. doi:10.1038/srep43461

Zhang, D., Zhang, Z., Li, J., Chang, Y., Du, Q., & Pan, T. (2015). Regulation of vapor pressure deficit by greenhouse micro-fog systems improved growth and productivity of tomato via enhancing photosynthesis during summer season. *PLOS ONE*, *10*(7), e0133919. doi:10.1371/journal.pone.0133919
